# Brachiopoda collection of the Shirshov Institute of Oceanology, Russian Academy of Sciences

**DOI:** 10.3897/BDJ.14.e191347

**Published:** 2026-06-24

**Authors:** Zoya Dudnik

**Affiliations:** 1 Shirshov Institute of Oceanology, Russian Academy of Sciences, Moscow, Russia Shirshov Institute of Oceanology, Russian Academy of Sciences Moscow Russia

**Keywords:** biodiversity dataset, biogeography, marine fauna, taxonomy, zoological collections

## Abstract

**Background:**

This dataset provides information for 3235 Brachiopoda specimens held within the Ocean Benthic Fauna collection (collection code: OBFc) in the Shirshov Institute of Oceanology (IORAS). Collected from 1852 unique locations across the World Ocean, these specimens represent a 100-year record of Brachiopoda biodiversity, with sampling beginning in 1924.

The IORAS Brachiopoda collection, due to its high species diversity, is a valuable resource for researchers. Broad taxonomic representation provides critical baseline data that fundamentally advance our understanding of brachiopod distribution patterns, ecological niches, and historical biogeography. The collection thus directly supports a wide range of disciplinary research, including taxonomy and phylogenetics, and the study of large-scale macroecological patterns in global marine ecosystems.

**New information:**

A comprehensive revision and digitization of the complete Brachiopoda collection of the Shirshov Institute of Oceanology has yielded a detailed dataset on specimen distribution across geographic, bathymetric, and taxonomic categories. The project prioritized the documentation and imaging of the type specimens. This new dataset is a significant scientific resource for taxonomy, biodiversity, and biogeography; this data enhances our understanding of marine biodiversity and the distribution patterns of Brachiopoda throughout various oceanic regions and depth zones.

## Introduction

### The IORAS Brachiopoda collection

The IORAS Brachiopoda collection was originally curated by Olga Nikolaevna Zezina (1937–2013), a famous Soviet and Russian zoologist and hydrobiologist, and a specialist in modern brachiopods. She has formed one of the world's largest collections of modern brachiopods. She has analyzed their habitats on continental slopes, islands, and underwater elevations and for the first time has proposed a scheme of biogeographical zoning of the ocean bathyal zone (Zezina 1970a, Zezina 1973, Zezina 1997a, Zezina 2008). The role of brachiopods as indicators of the natural bottom biofilter of the ocean deeper than the photic layer has been proven (Zezina 1985, Zezina 1997b). Based on the results of processing mass deep-sea collections, she has obtained for the first time empirical data on the ratio of production and biomass of bottom invertebrates.

While O. N. Zezina was a foremost expert on deep-sea brachiopod ecology and biogeography, she had also described the new brachiopod family Tythothyrididae Zezina, 1979 (Zezina 1979) (not accepted in the new edition of Treatise on Invertebrate Paleontology); two subfamilies – Acanthobasiliolinae Zezina, 1981 and Phaneroporinae Zezina, 1981 (Zezina 1981a); 11 new genera; 21 new species, and 3 new subspecies. The collection houses 72 type Brachiopoda specimens, representing 18 species and 2 subspecies (Table 1).

The digitization of the IORAS Brachiopoda collection was conducted utilizing Specify 6 software (Specify Collections Consortium 2023). The digital catalogue features scientific names (standardized via WoRMS (WoRMS Editorial Board 2025)), collection dates, geographic coordinates, depth, images, and other relevant data for every lot. Subsequently, this catalogue was converted into a Darwin Core occurrence dataset and made publicly available on the Global Biodiversity Information Facility (GBIF) (Dudnik 2025).

## Sampling methods

### Sampling description

**Figure 11.**
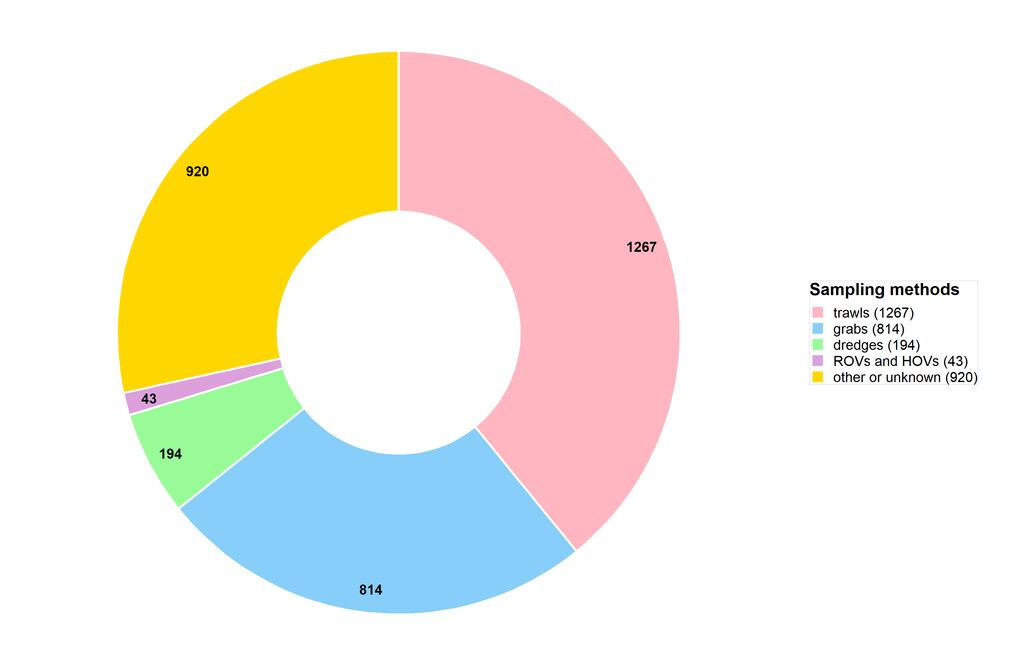
Collection methods of the IORAS Brachiopoda specimens. Numbers correspond to the amount of collection lots.

The collection specimens were mainly obtained during the research cruises using Sigsbee trawls (779 lots), "Okean" grabs (699 lots), dredges (194 lots), and bottom and Agassiz trawls. In total, 1267 brachiopod collection lots (69% of the whole collection) were obtained by different trawl types (Fig. 11).

## Geographic coverage

### Description

**Figure 12.**
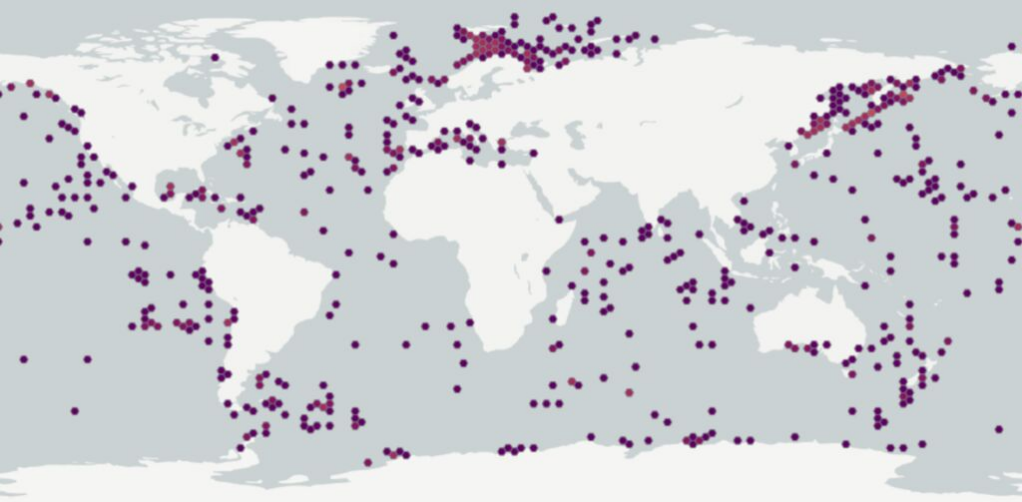
Sampling localities of IORAS brachiopods (GBIF.org 2025).

**Figure 13.**
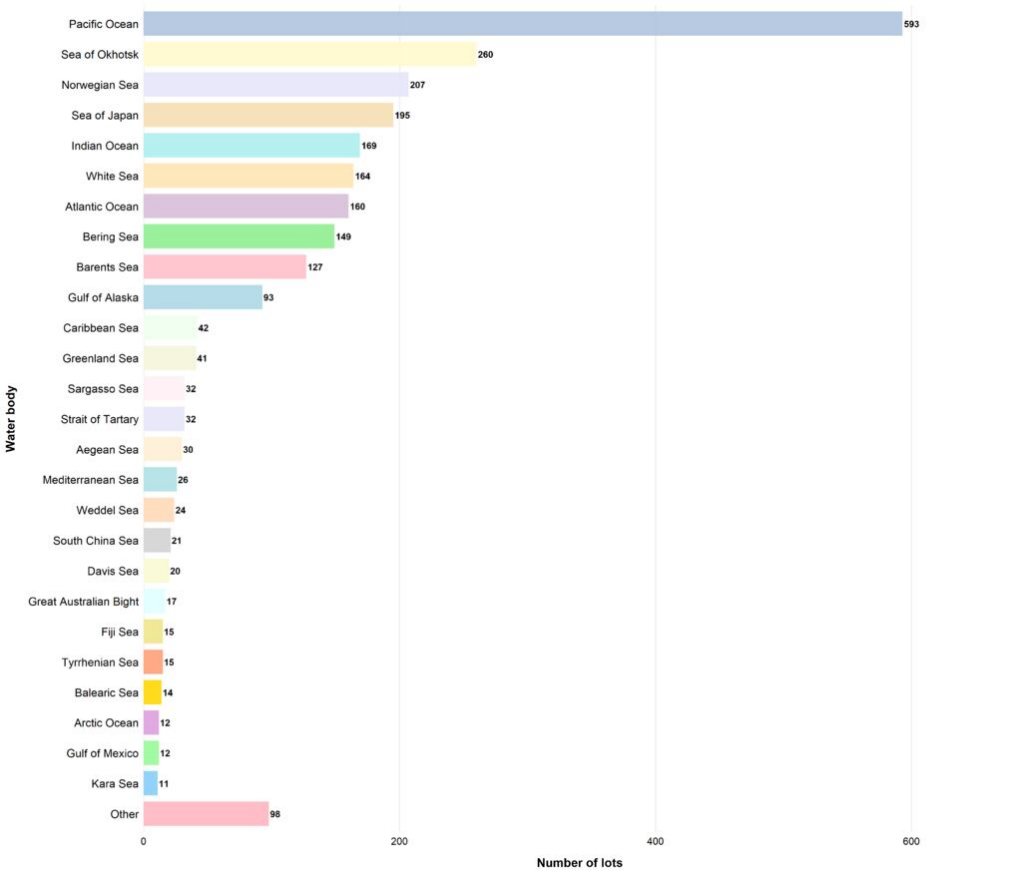
Geographic origin of brachiopods deposited in the IORAS collection. Numbers correspond to the amount of collection lots.

This collection features samples collected from 1924 to today from 1825 locations in the World Ocean (Fig. 12), based on 248 research voyages. It is especially rich in specimens from the Arctic and Northwest Pacific. Most were gathered by Soviet and Russian research vessels (including the Vityaz, Krylatka, Nikolay Maslov, Atna, and Akademik Kurchatov), specifically from the Sea of Okhotsk, Norwegian Sea, and Sea of Japan (Fig. 13).

**Figure 14.**
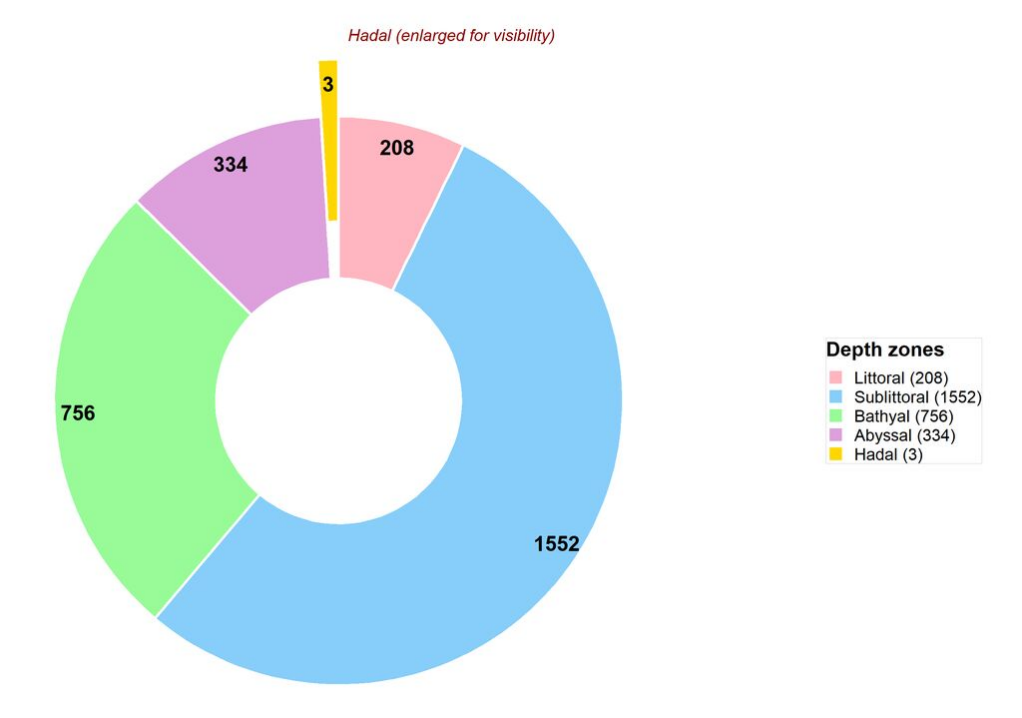
Bathymetric distribution of IORAS brachiopods. Numbers correspond to the amount of collection lots; sector"Hadal" is enlarged for visibility.

Specimens were collected across all ocean depth zones (Fig. 14). The collection is particularly rich in deep-sea fauna, containing 756 lots from the bathyal zone and 334 from the abyssal zone. It also includes three rare specimens collected from the ultra-abyssal (hadal) zone of the Romanche Trench in the Northwest Pacific (Table 2).

## Taxonomic coverage

### Description

**Figure 15.**
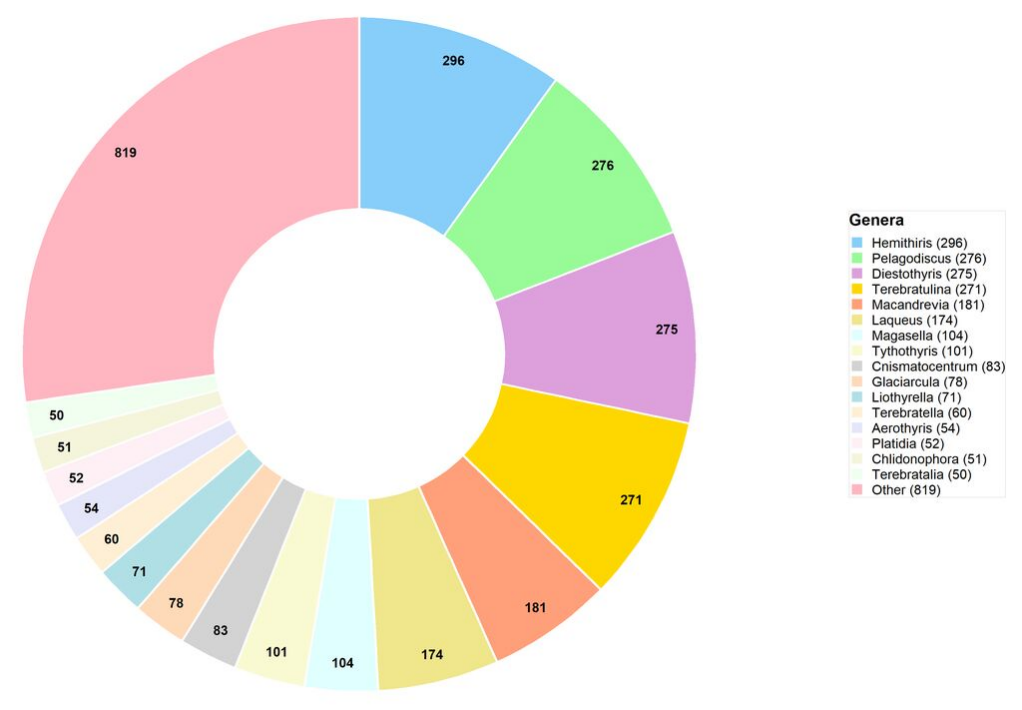
Most represented genera by number of lots (shown in numbers) in the IORAS Brachiopoda collection.

**Figure 16.**
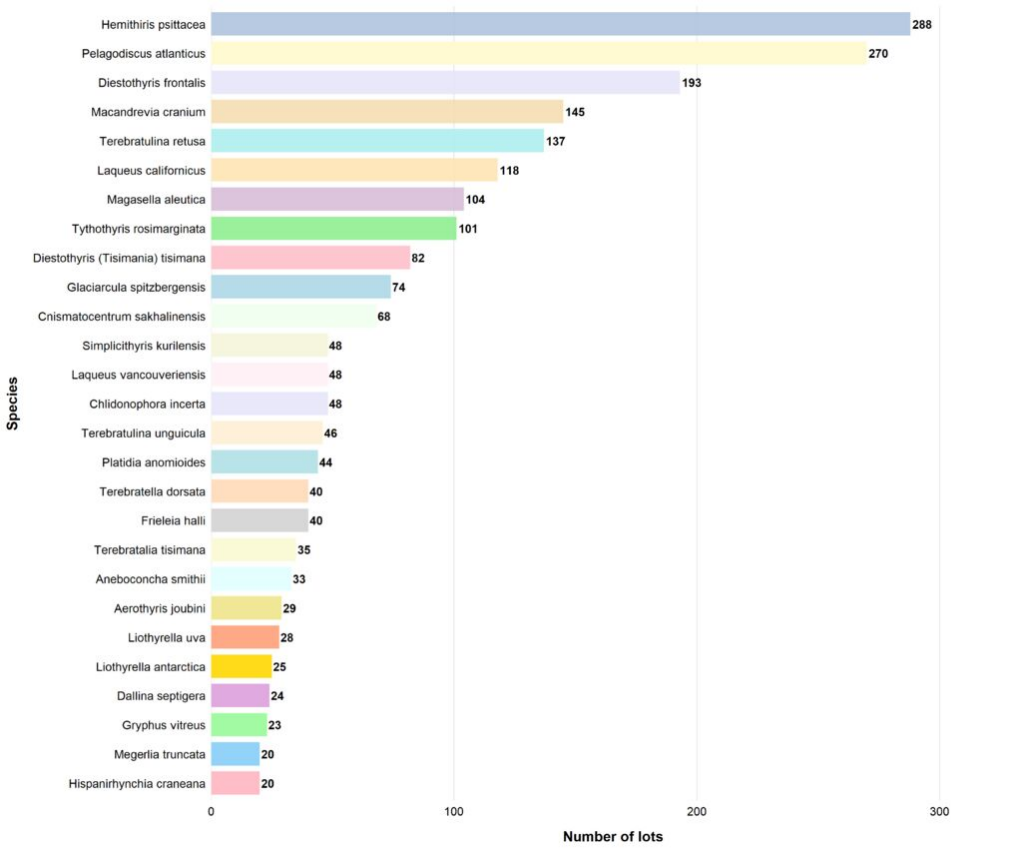
Most represented species by number of lots (shown in numbers) in the IORAS Brachiopoda collection.

A total of 88% of the collection specimens are identified to the species level. The collection includes 173 species belonging to 96 genera and 30 families of Brachiopoda (Fig. 15, Fig. 16, Table 3).

### Taxa included

**Table:** 

Rank	Scientific Name	
family	Aulacothyropsidae	
family	Basiliolidae	
family	Cancellothyrididae	
family	Chlidonophoridae	
family	Cnismatocentridae	
family	Craniidae	
family	Cryptoporidae	
family	Dallinidae	
family	Discinidae	
family	Dyscoliidae	
family	Frenulinidae	
family	Frieleiidae	
family	Hemithirididae	
family	Jagtithyrididae	
family	Kraussinidae	
family	Laqueidae	
family	Megathyrididae	
family	Notosariidae	
family	Obolidae	
family	Phaneroporidae	
family	Platidiidae	
family	Rhynchonellidae	
family	Terebrataliidae	
family	Terebratellidae	
family	Terebratulidae	
family	Tethyrhynchiidae	
family	Thaumatosiidae	
family	Thecideidae	
family	Zeilleriidae	

## Temporal coverage

**Data range:** 1924-10-03 – 2024-8-11.

## Collection data

### Collection name

Ocean Benthic Fauna collection

### Collection identifier

OBFc

### Specimen preservation method

Ethanol, dried

### Curatorial unit

Laboratory of Ocean Benthic Fauna

## Usage licence

### Usage licence

Other

### IP rights notes

Creative Commons Attribution Non-Commercial (CC-BY-NC) 4.0 Licence

## Data resources

### Data package title

Brachiopoda collection of the Shirshov Institute of Oceanology, Laboratory of Ocean Benthic Fauna

### Resource link

https://doi.org/10.15468/k9x9ph

### Alternative identifiers

https://www.gbif.org/dataset/d62e2c55-68e7-41d1-9dc0-c7f1bb1d45b6

### Number of data sets

1

### Data set 1.

#### Data set name

Brachiopoda collection of the Shirshov Institute of Oceanology, Laboratory of Ocean Benthic Fauna

#### Data format

Darwin Core Archive

#### Character set

UTF-8

#### Download URL

https://gbif.ocean.ru/ipt/archive.do?r=brachiopoda_ioras&v=1.19

#### Data format version

1.2

#### Description

The dataset contains data on the Brachiopoda specimens stored in the Ocean Benthic Fauna collection of the Shirshov Institute of Oceanology (IORAS).

**Data set 1.** 

Column label	Column description
occurrenceID	An identifier for the dwc:Occurrence (as opposed to a particular digital record of the dwc:Occurrence).
institutionID	An identifier for the institution having custody of the object(s) or information referred to in the record.
collectionID	An identifier for the collection from which the record was derived.
institutionCode	The acronym in use by the institution having custody of the object(s) or information referred to in the record.
collectionCode	The acronym identifying the collection from which the record was derived.
ownerInstitutionCode	The acronym in use by the institution having ownership of the object(s) or information referred to in the record.
basisOfRecord	The specific nature of the data record.
catalogNumber	An identifier for the record within the collection.
eventRemarks	Name of RV (research vessel) on board which the original dwc:Occurrence recording was made, and a remark if cruise number is unknown.
individualCount	The number of individuals present at the time of the dwc:Occurrence.
occurrenceStatus	A statement about the presence or absence of a dwc:Taxon at a dcterms:Location.
preparations	A preparation or preservation method for a specimen. Such as dried, EtOH 75% (stored in 75% ethanol), SEM (dehydrated, mounted onto a stub and sputter-coated with a conductive material for scanning electron microscopy), slide (microscope slide), dried:EtOH 75% (stored in 75% ethanol, then dried)
associatedMedia	A list of URLs of media associated with the dwc:Occurrence.
eventID	An identifier for the set of information associated with a dwc:Event in the format "RV name_cruise number_st.number".
parentEventID	An identifier for the RV name and the cruise number where the original dwc:Occurrence was recorded.
fieldNumber	An identifier for the station number where the original dwc:Occurrence was recorded.
eventDate	The date when the dwc:Event was recorded.
samplingProtocol	The names of the methods or protocols used during a dwc:Event. Such as trawls (Sigsbee, Agassiz, Galathea etc.), dredges, grabs (common - DO ("Okean" grab), DG (Gorbunov grab), DP (Peterson grab), and television-guided TV grabs), box corers (common and television-guided TV multicorers), submersibles (HOVs (human occupied vehicles) Mir-1, Mir-2 and Pisces and ROVs (remotely operated vehicles).
waterBody	The name of the water body in which the dcterms:Location occurs.
islandGroup	The name of the island group in which the dcterms:Location occurs.
country	The name of the country or major administrative unit in which the dcterms:Location occurs.
countryCode	The standard code for the country in which the dcterms:Location occurs.
locality	The original textual description of the place.
verbatimDepth	The original description of the depth below the local surface. Range means depths of start and end of sampling.
decimalLatitude	The geographic latitude (in decimal degrees, using the spatial reference system given in dwc:geodeticDatum) of the geographic centre of a dcterms:Location. Positive values are north of the Equator, negative values are south of it.
decimalLongitude	The geographic longitude (in decimal degrees, using the spatial reference system given in dwc:geodeticDatum) of the geographic centre of a dcterms:Location. Positive values are east of the Greenwich Meridian, negative values are west of it.
geodeticDatum	The ellipsoid, geodetic datum or spatial reference system (SRS), upon which the geographic coordinates given in dwc:decimalLatitude and dwc:decimalLongitude are based.
taxonRank	The taxonomic rank of the most specific name in the dwc:scientificName.
typeStatus	Nomenclatural type (type status) applied to the subject.
identifiedBy	Person name who assigned the dwc:Taxon to the subject.
scientificName	The name in lowest level taxonomic rank that can be determined.
nameAccordingTo	The reference to the source in which the specific taxon concept circumscription is defined or implied.
kingdom	The full scientific name of the kingdom in which the dwc:Taxon is classified.
phylum	The full scientific name of the phylum or division in which the dwc:Taxon is classified.
class	The full scientific name of the class in which the dwc:Taxon is classified.
order	The full scientific name of the order in which the dwc:Taxon is classified.
family	The full scientific name of the subfamily in which the dwc:Taxon is classified.
genus	The full scientific name of the genus in which the dwc:Taxon is classified.

## Figures and Tables

**Figure 1a.**
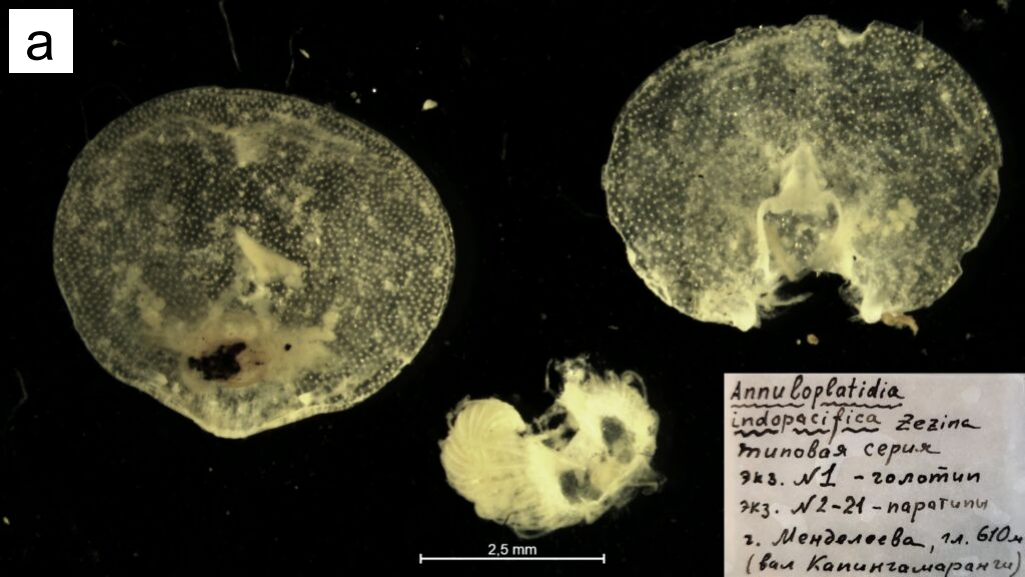
*Annuloplatidia indopacifica* holotype, cat. INV0008255

**Figure 1b.**
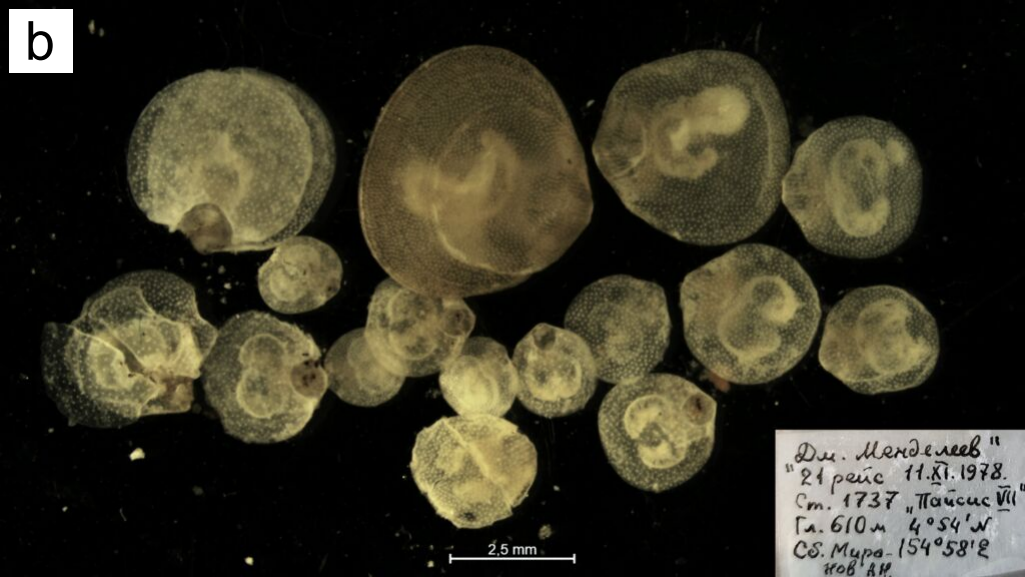
*Annuloplatidia indopacifica* paratypes, cat. INV0008256

**Figure 1c.**
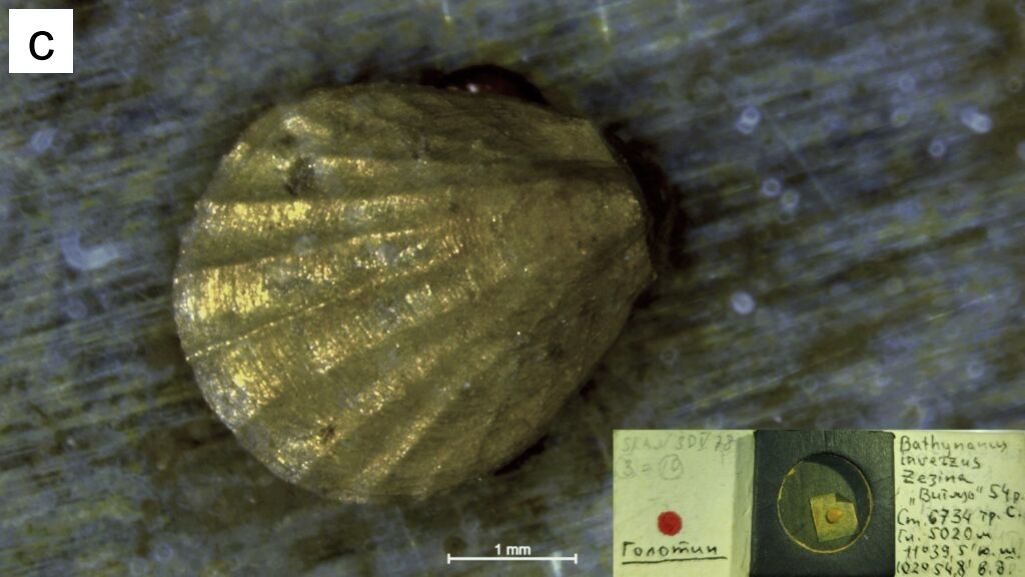
*Bathynanus inversus* holotype, cat. INV0003724

**Figure 1d.**
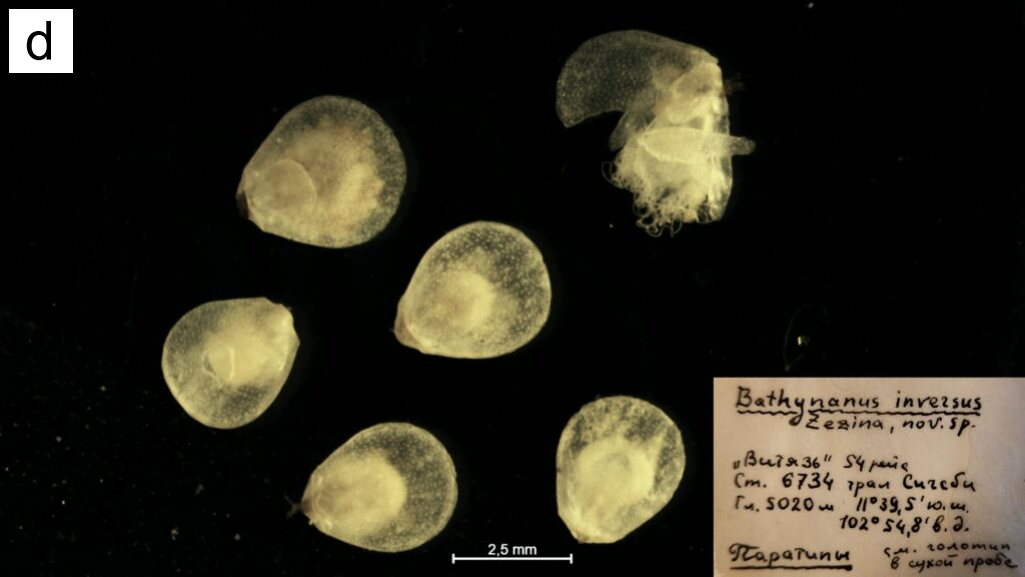
*Bathynanus inversus* paratypes, cat. INV0006022

**Figure 1e.**
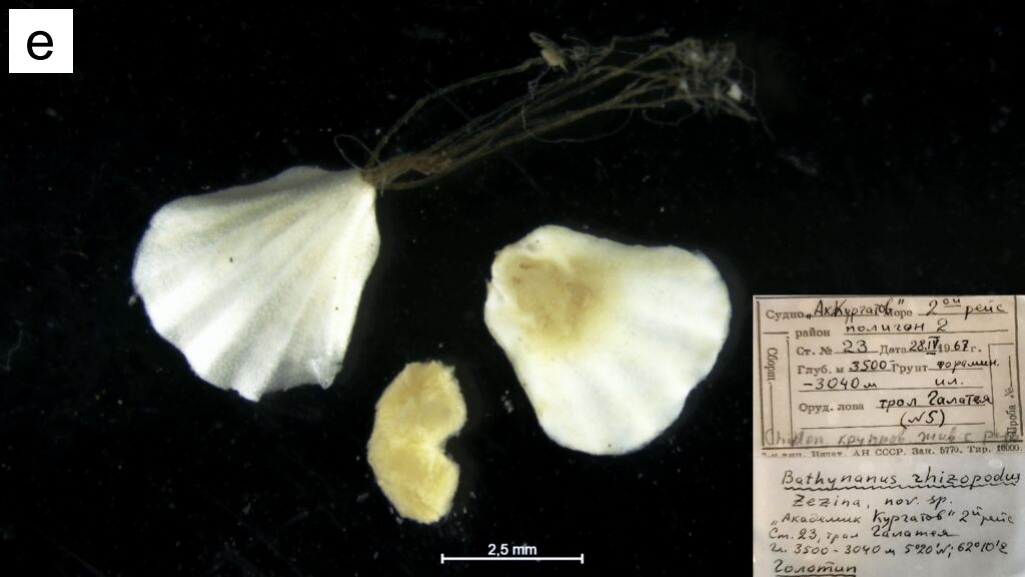
*Bathynanus rhizopodus* holotype, cat. INV0003719

**Figure 1f.**
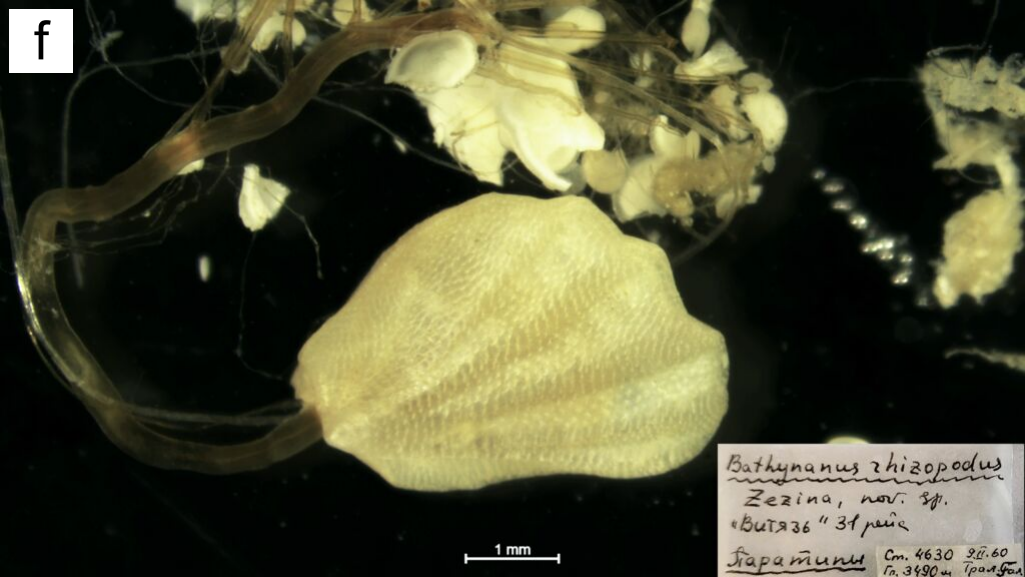
*Bathynanus rhizopodus* paratype, cat. INV0003720

**Figure 2a.**
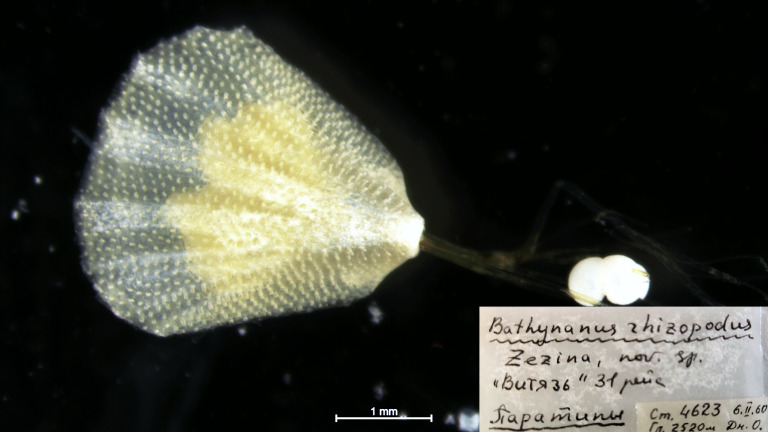
*Bathynanus rhizopodus* paratype, cat. INV0003721

**Figure 2b.**
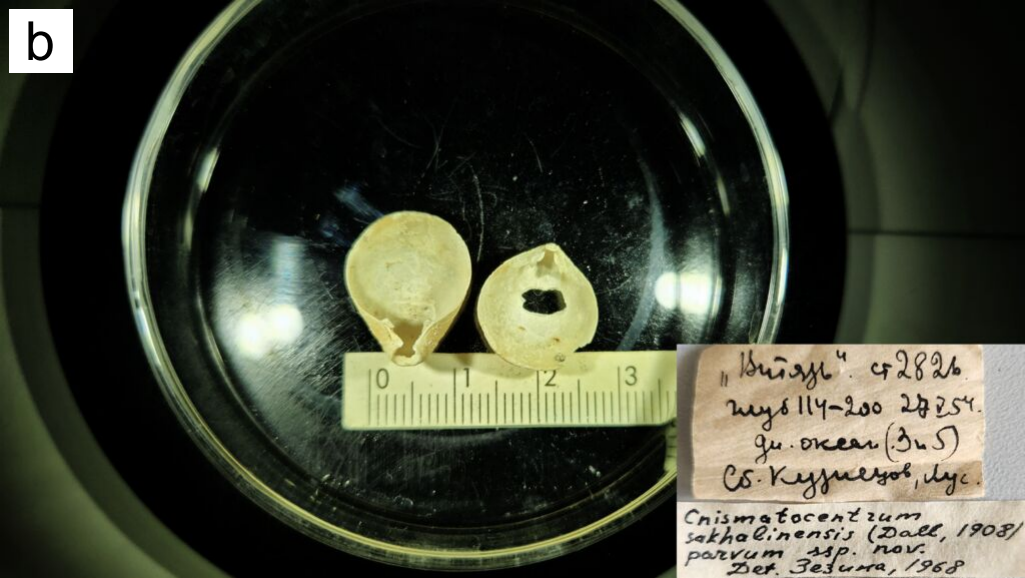
*Cnismatocentrum sakhalinensis parvum* syntype, cat. INV0006016

**Figure 2c.**
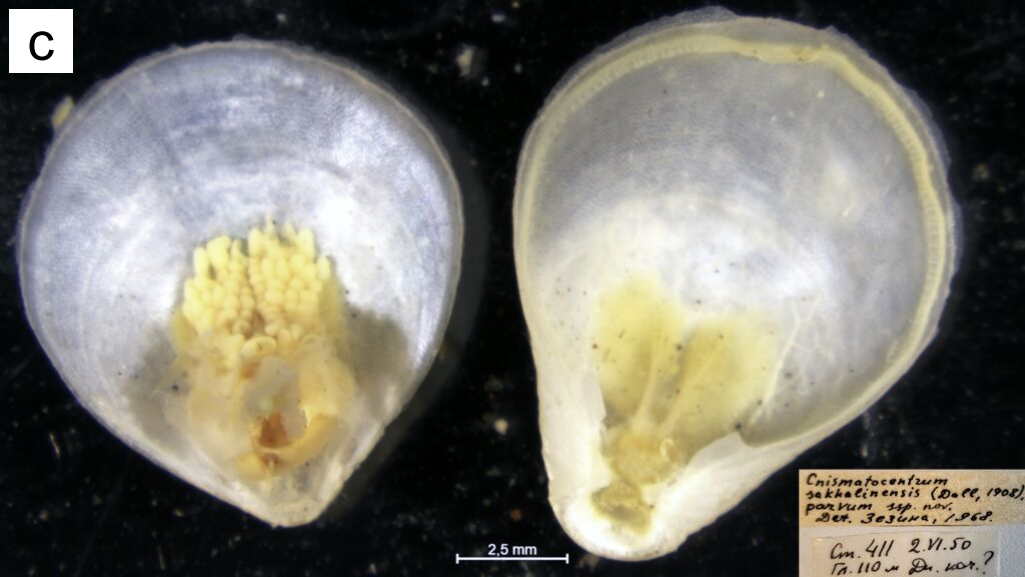
*Cnismatocentrum sakhalinensis parvum* syntype, cat. INV0006018

**Figure 2d.**
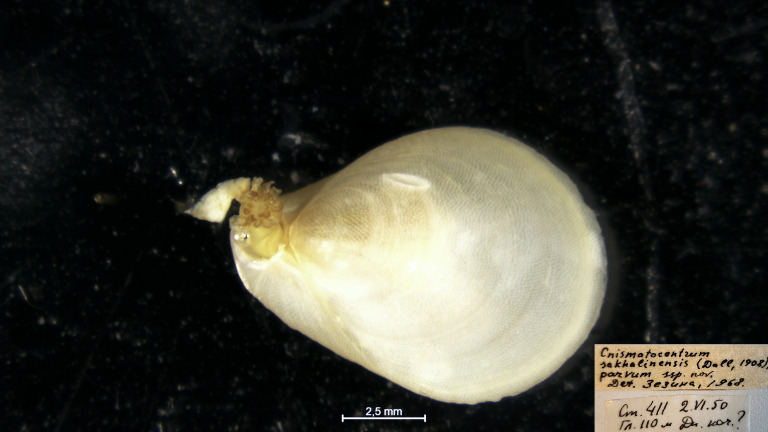
*Cnismatocentrum sakhalinensis parvum* syntype, cat. INV0006018

**Figure 2e.**
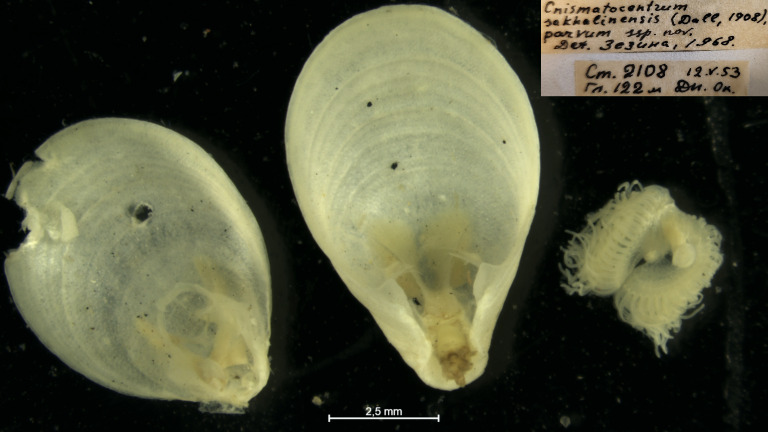
*Cnismatocentrum sakhalinensis parvum* syntype, cat. INV0006019

**Figure 2f.**
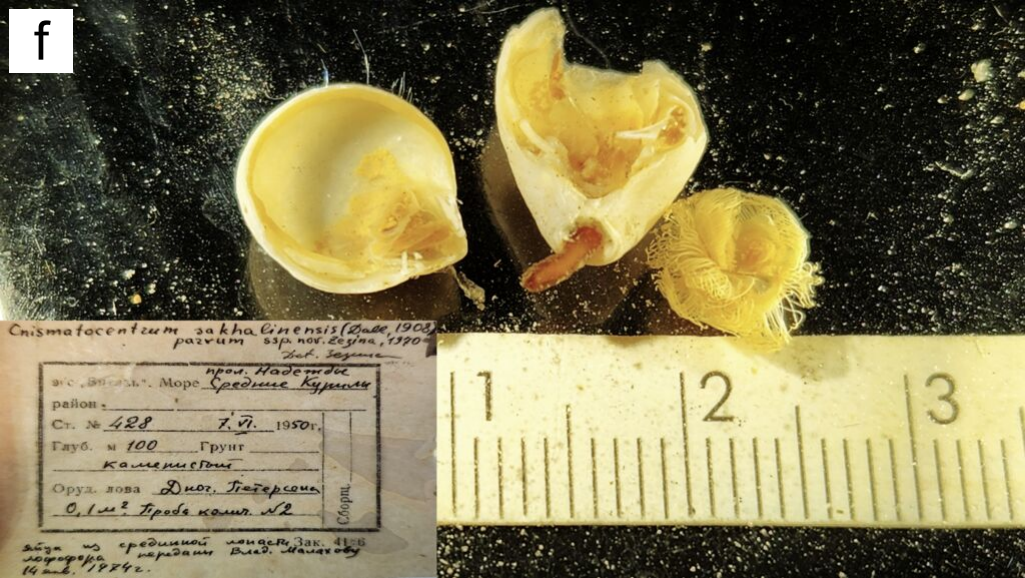
*Cnismatocentrum sakhalinensis parvum* syntype, cat. INV0006020

**Figure 3a.**
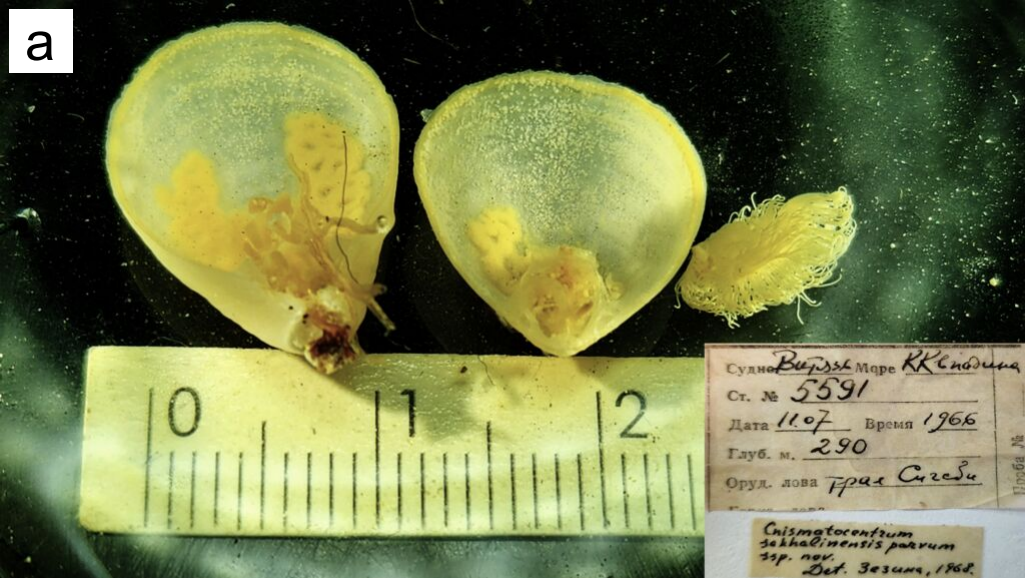
*Cnismatocentrum sakhalinensis parvum* syntype, cat. INV0006021

**Figure 3b.**
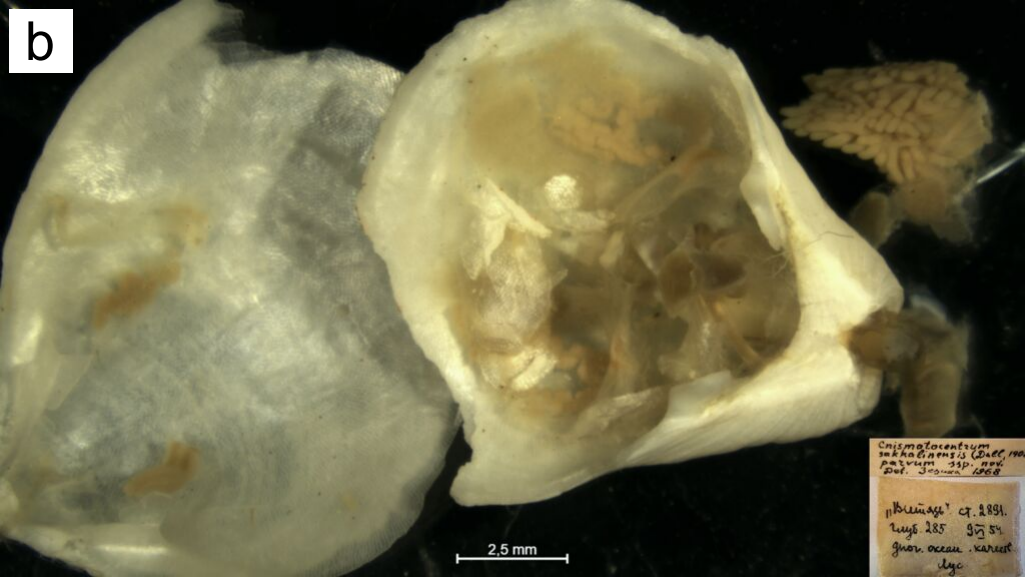
*Cnismatocentrum sakhalinensis parvum* syntype, cat. INV0006026

**Figure 3c.**
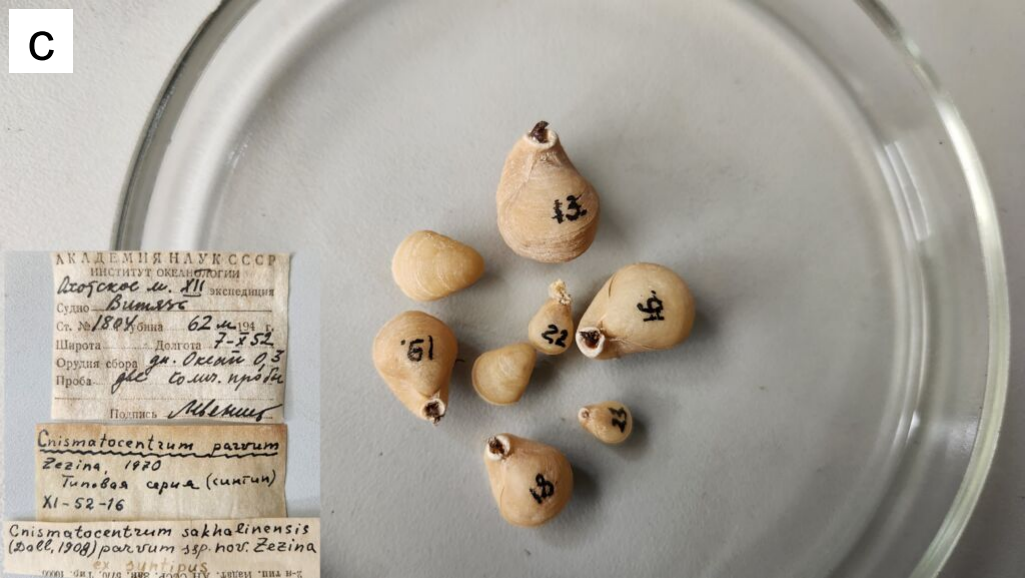
*Cnismatocentrum sakhalinensis parvum* syntype, cat. INV0009001

**Figure 3d.**
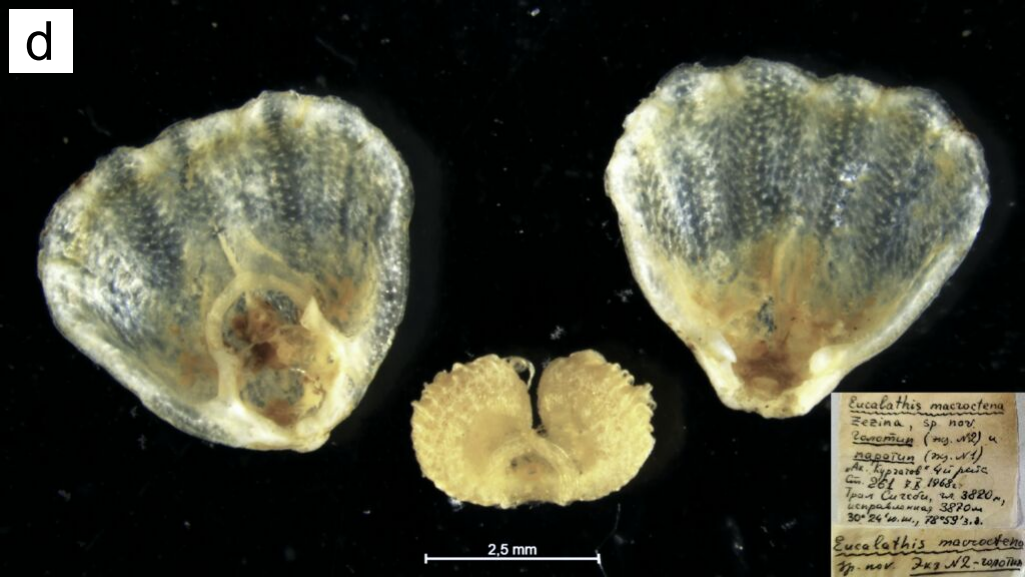
*Eucalathis macroctena* holotype, cat. INV0003713

**Figure 3e.**
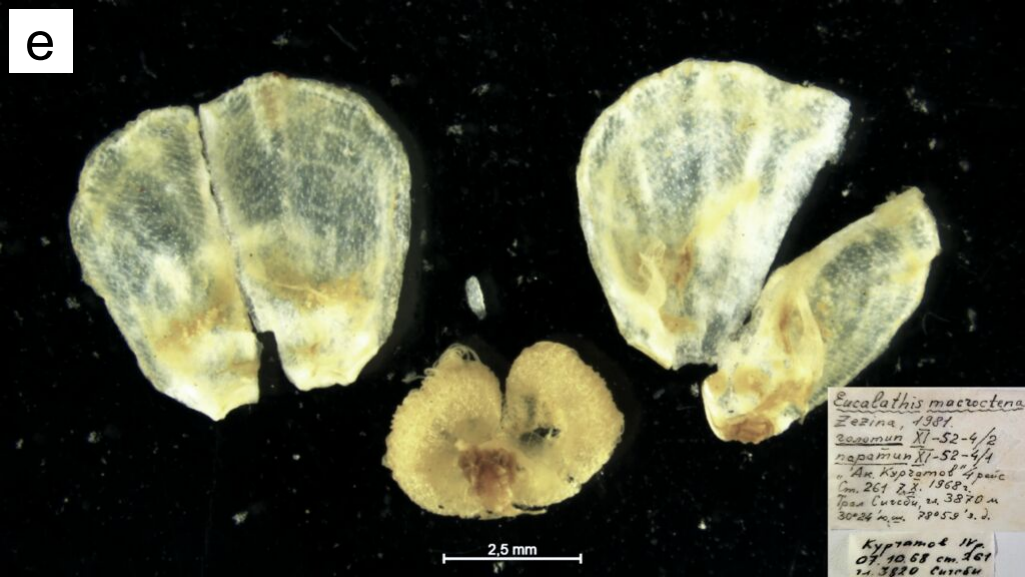
*Eucalathis macroctena* paratype, cat. INV0003714

**Figure 3f.**
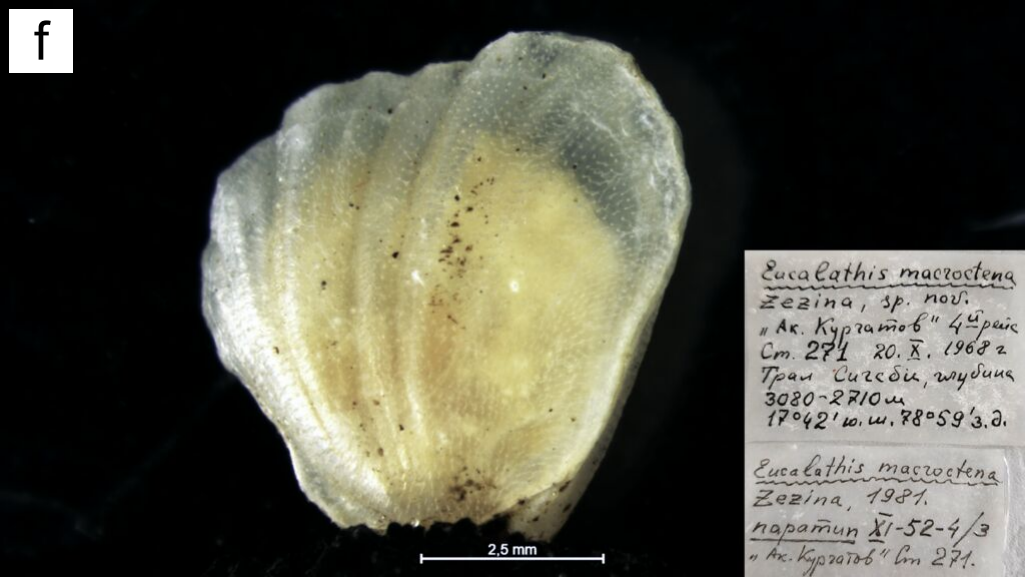
*Eucalathis macroctena* paratype, cat. INV0003715

**Figure 4a.**
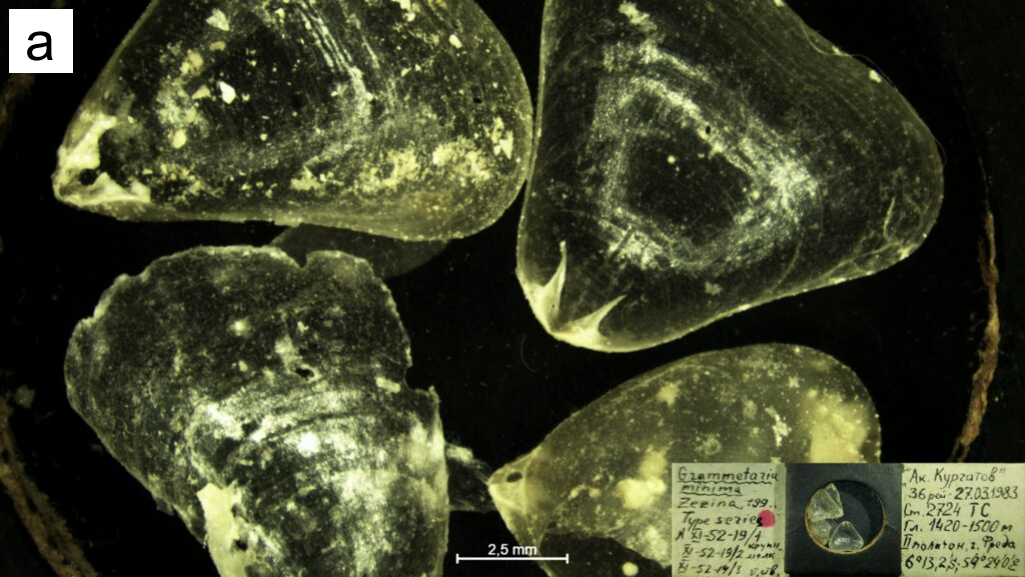
*Grammetaria minima* holotype and paratypes, cat. INV0008251 (further morphological study is required to differentiate them)

**Figure 4b.**
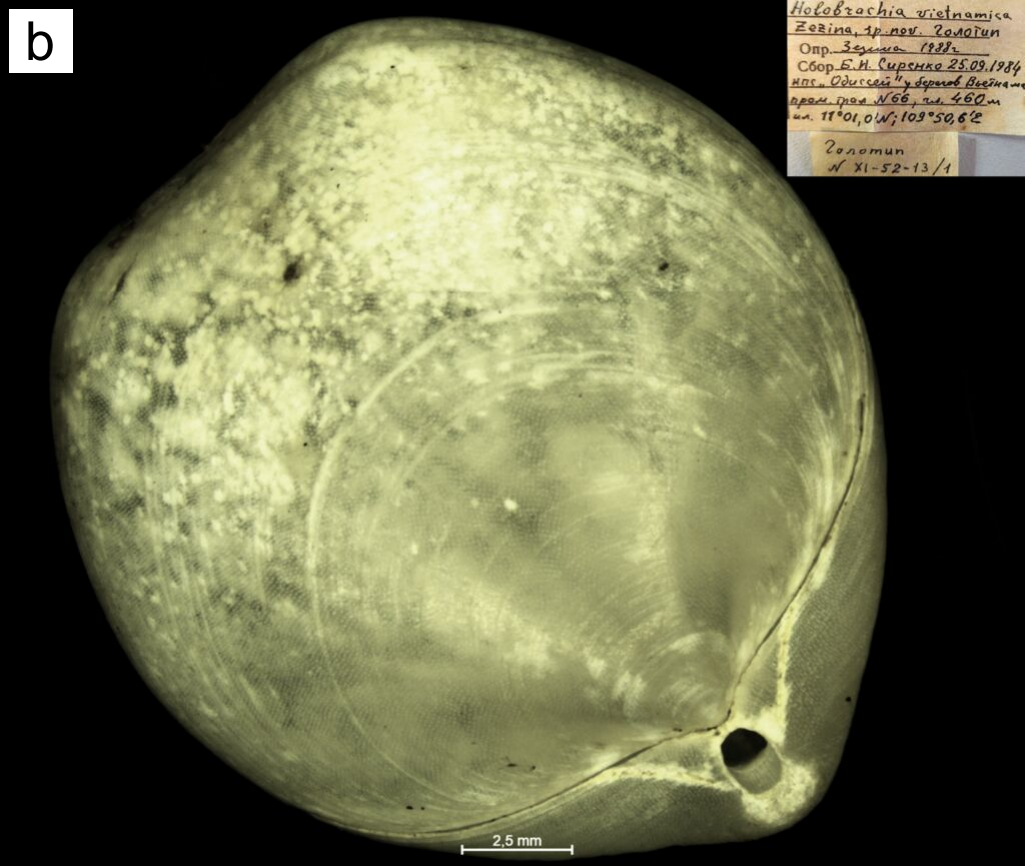
*Holobrachia vietnamica* holotype, cat. INV0008264

**Figure 4c.**
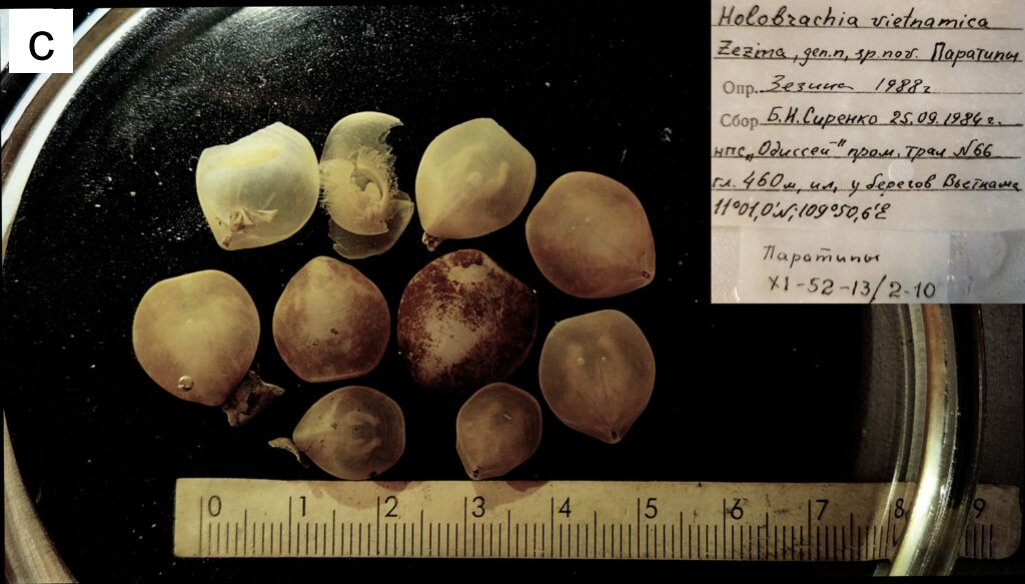
*Holobrachia vietnamica* paratypes, cat. INV0008267

**Figure 4d.**
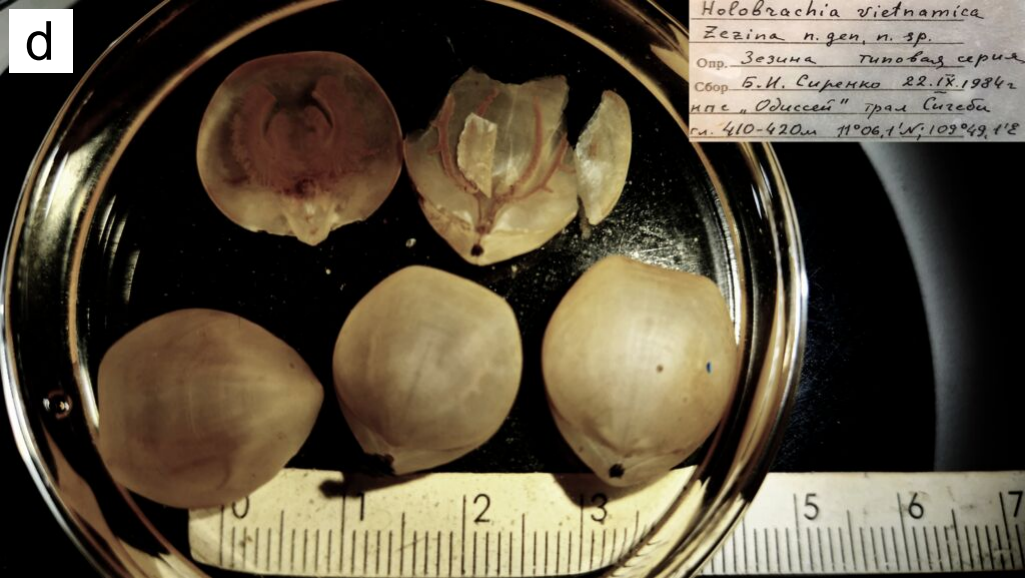
*Holobrachia vietnamica* topotypes, cat. INV0008265

**Figure 4e.**
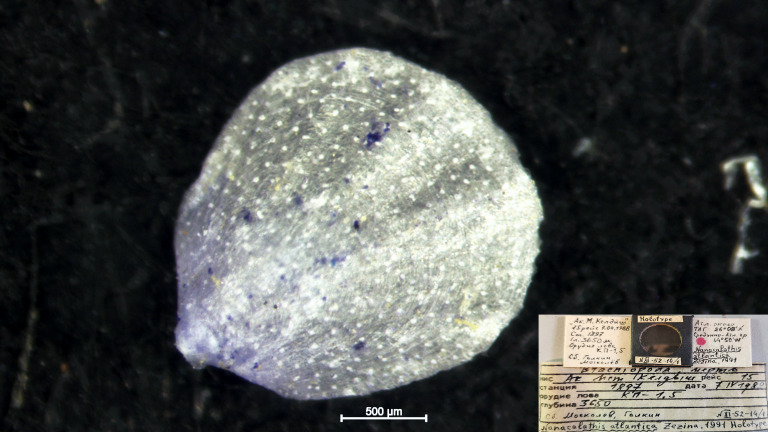
*Nanacalathis atlantica* holotype, cat. INV0003718

**Figure 4f.**
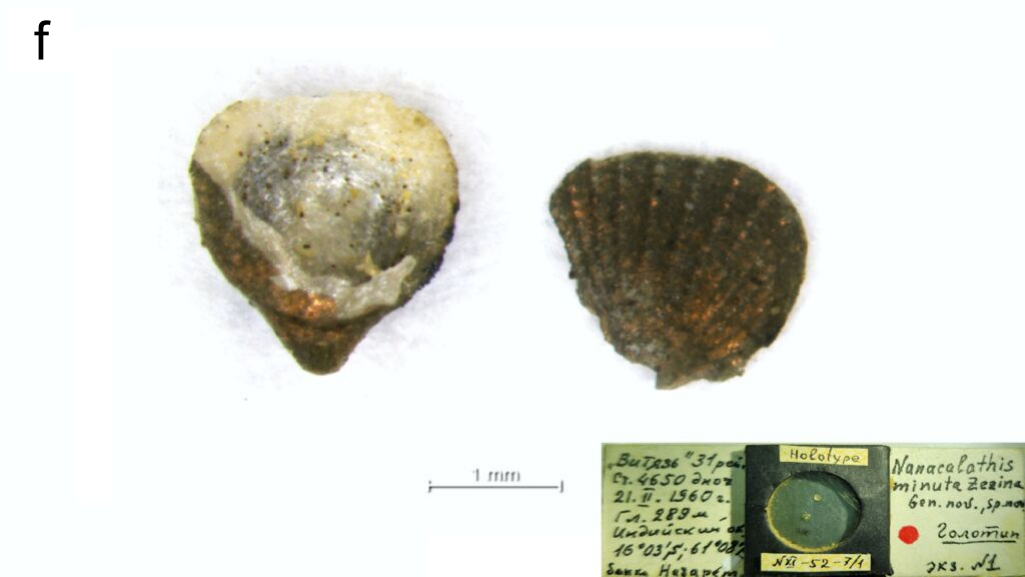
*Nanacalathis minuta* holotype, cat. INV0003716

**Figure 5a.**
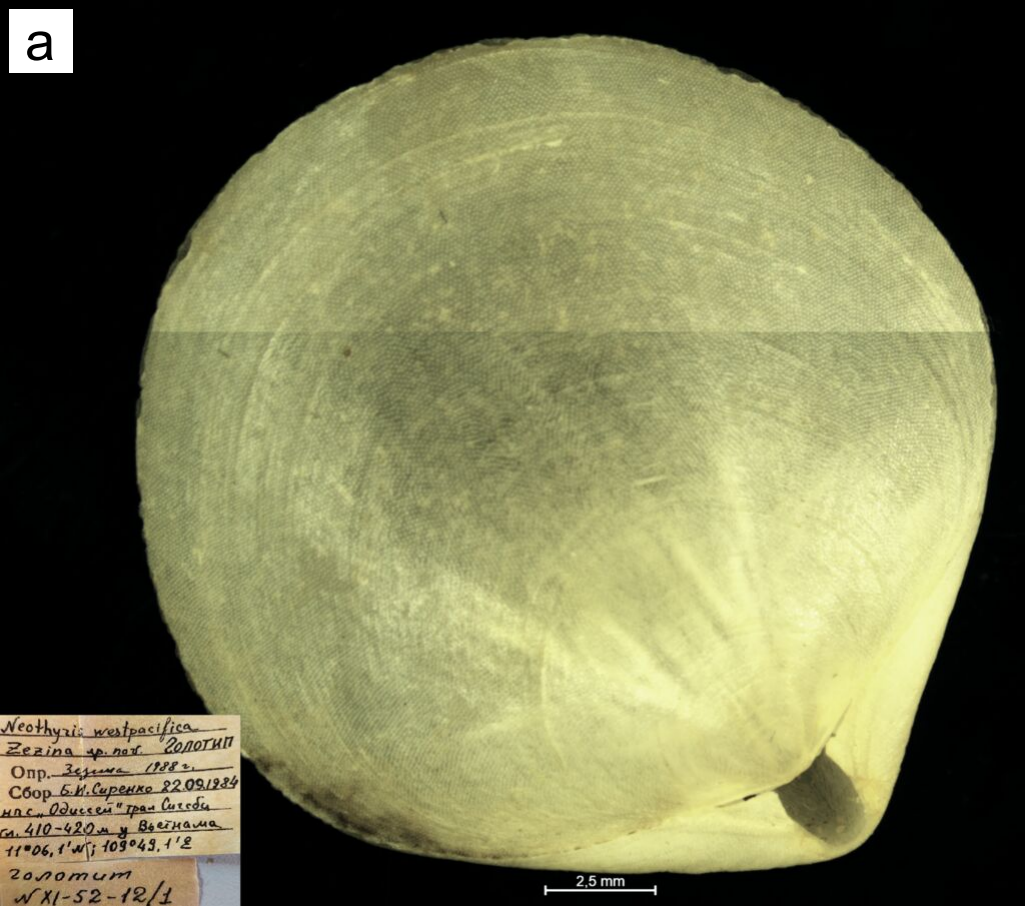
*Neothyris westpacifica* holotype, cat. INV0008259

**Figure 5b.**
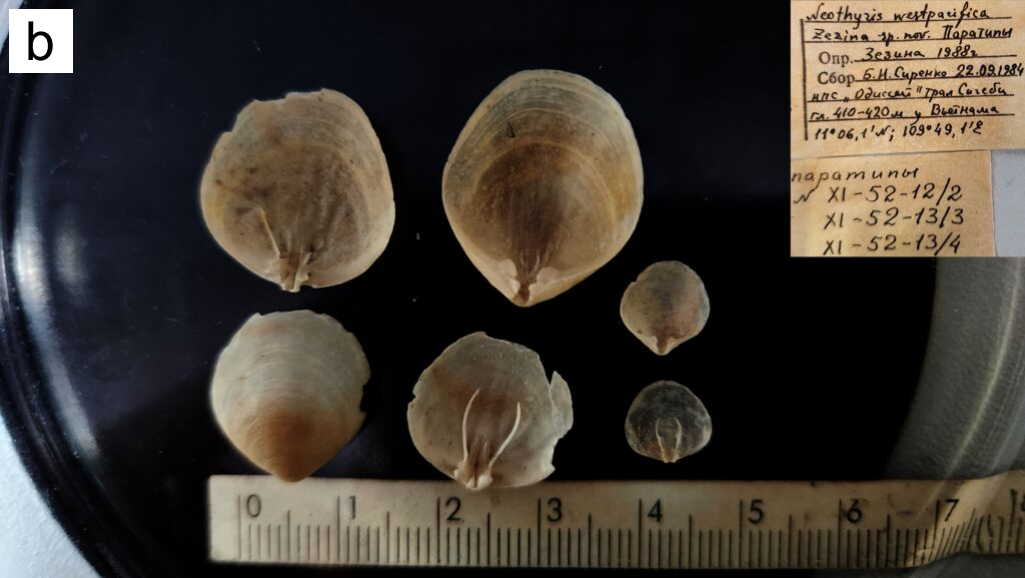
*Neothyris westpacifica* paratypes, cat. INV0008260

**Figure 5c.**
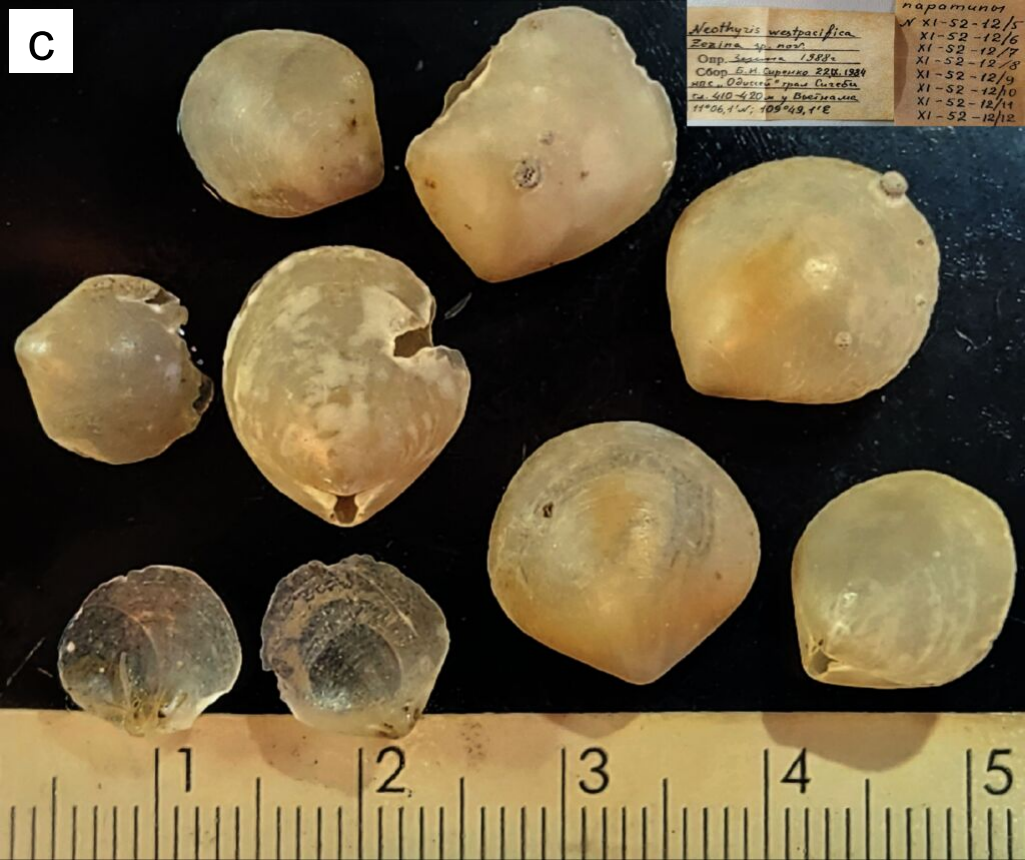
*Neothyris westpacifica* paratypes, cat. INV0008261

**Figure 5d.**
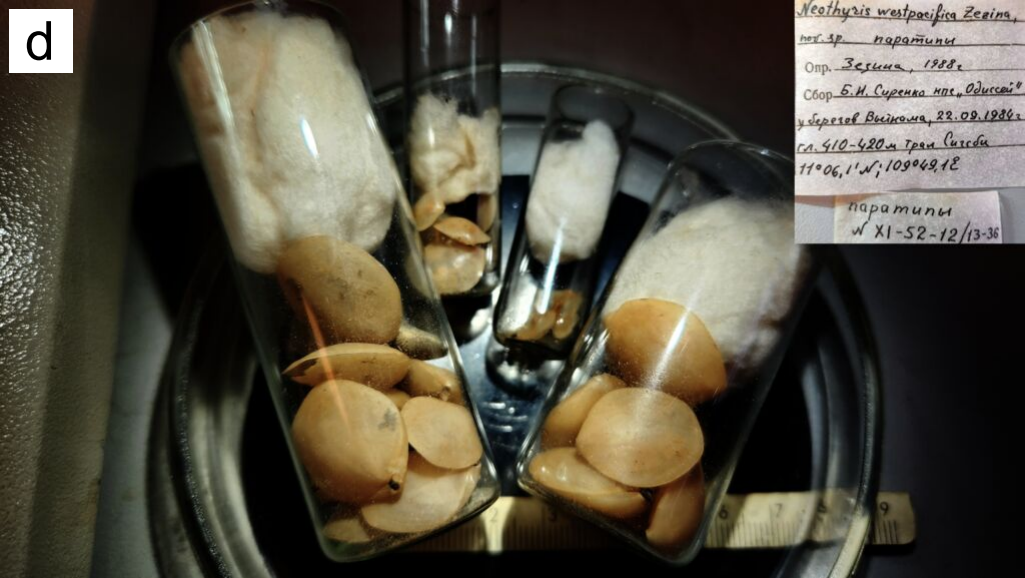
*Neothyris westpacifica* paratypes, cat. INV0008263

**Figure 5e.**
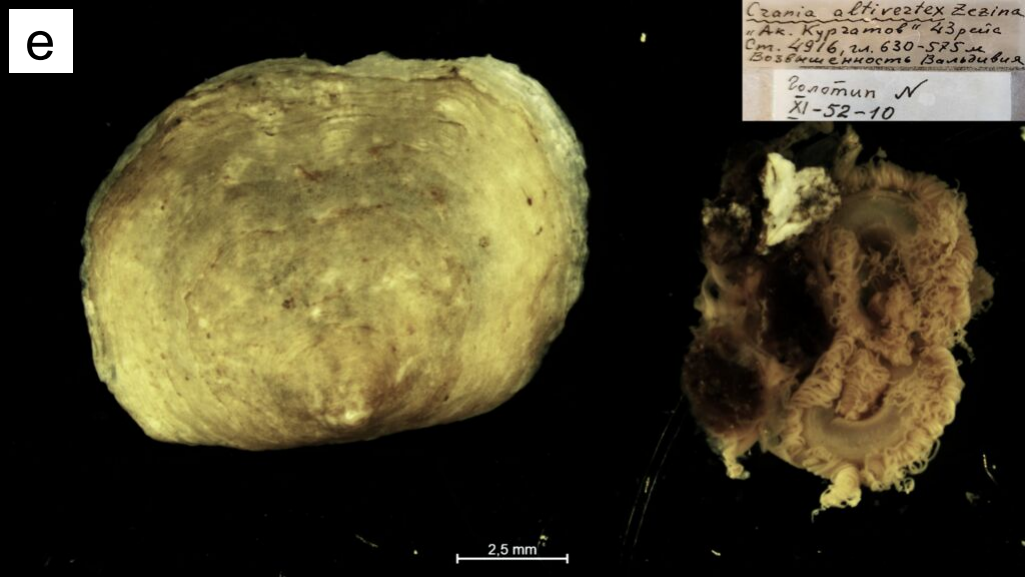
*Novocrania altivertex* holotype, cat. INV0008240

**Figure 5f.**
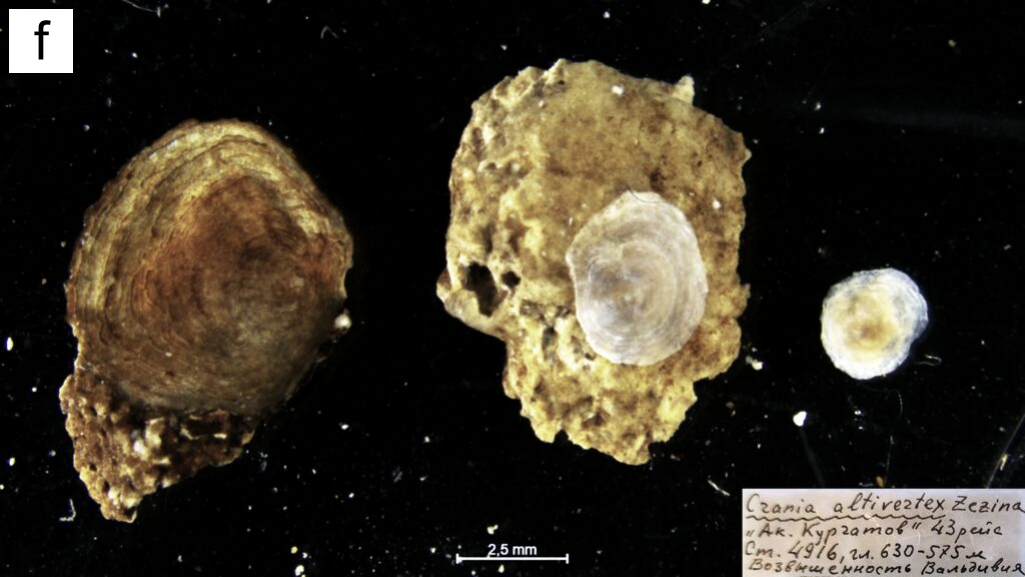
*Novocrania altivertex* paratypes, cat. INV0009026

**Figure 6a.**
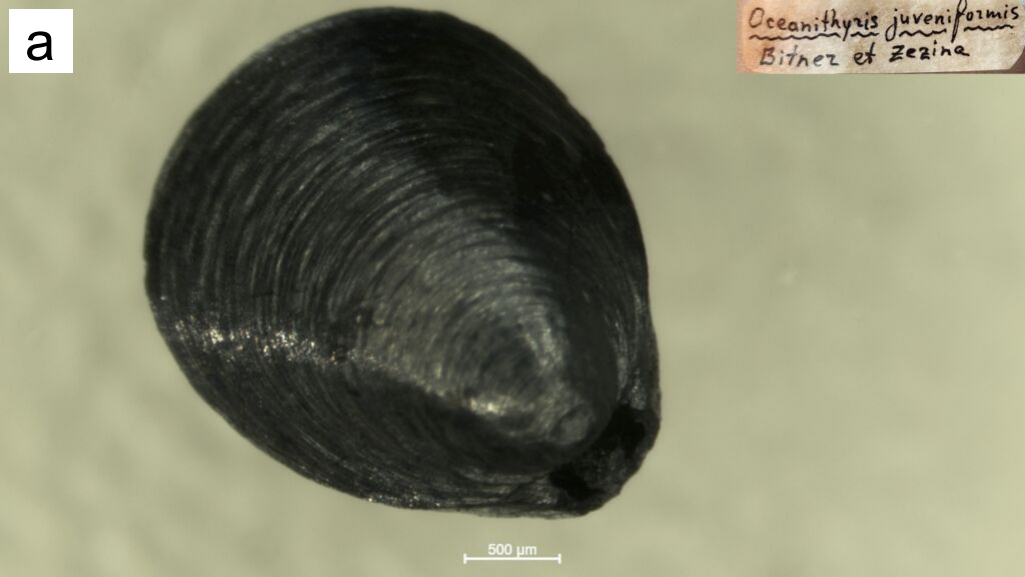
*Oceanithyris juveniformis* holotype, cat. INV0009005

**Figure 6b.**
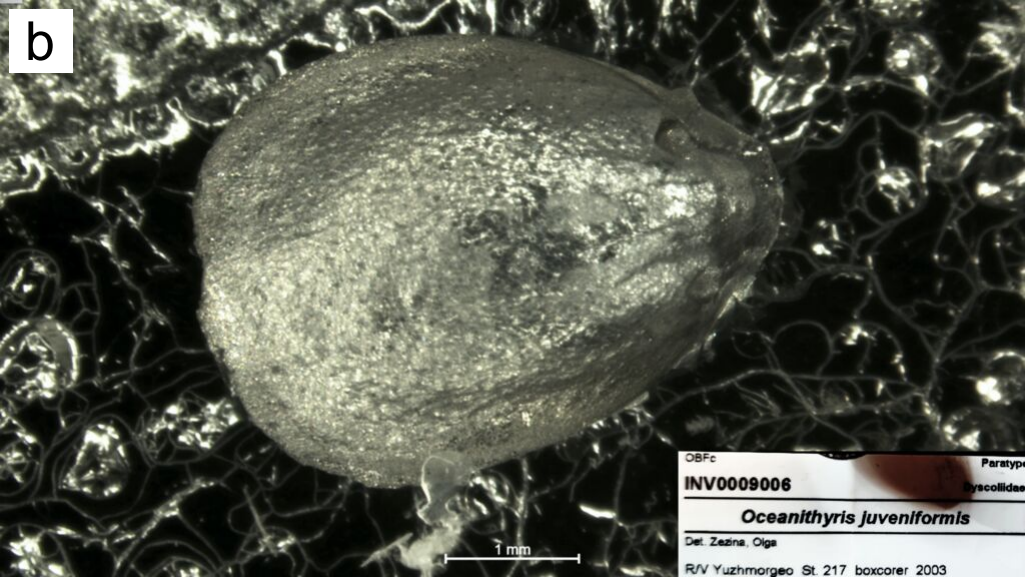
*Oceanithyris juveniformis* paratype, cat. INV0009006

**Figure 6c.**
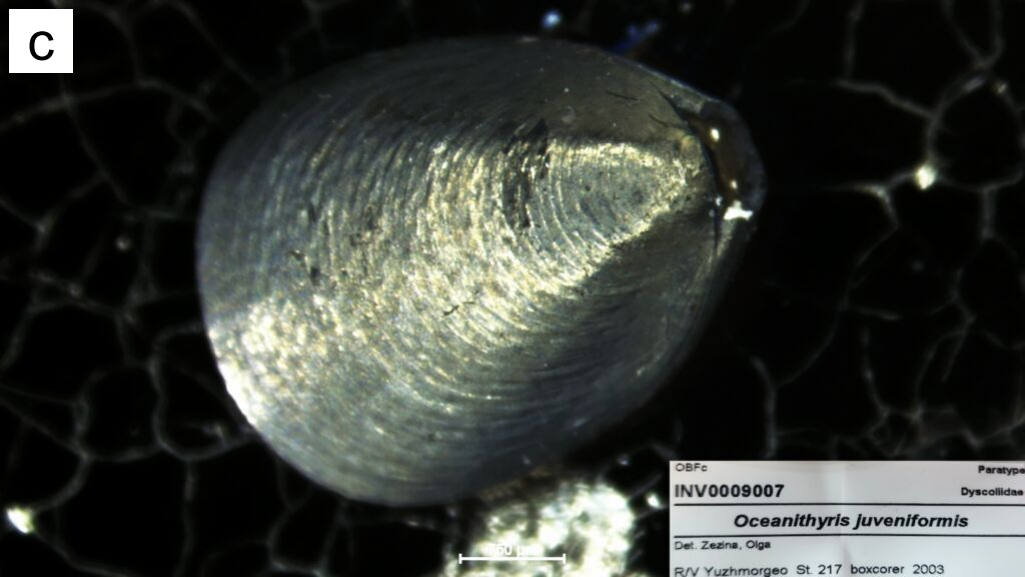
*Oceanithyris juveniformis* paratype, cat. INV0009007

**Figure 6d.**
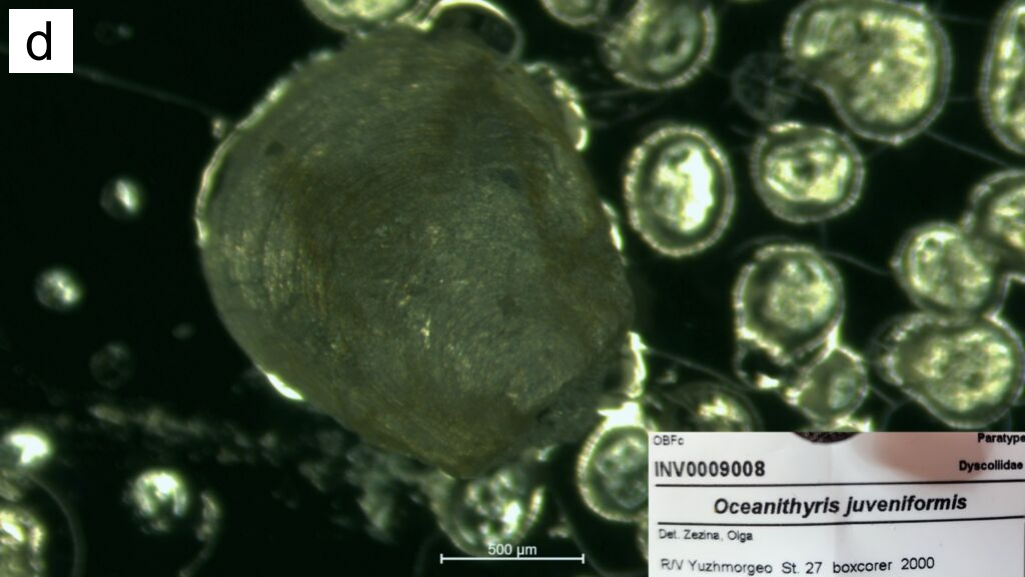
*Oceanithyris juveniformis* paratype, cat. INV0009008

**Figure 6e.**
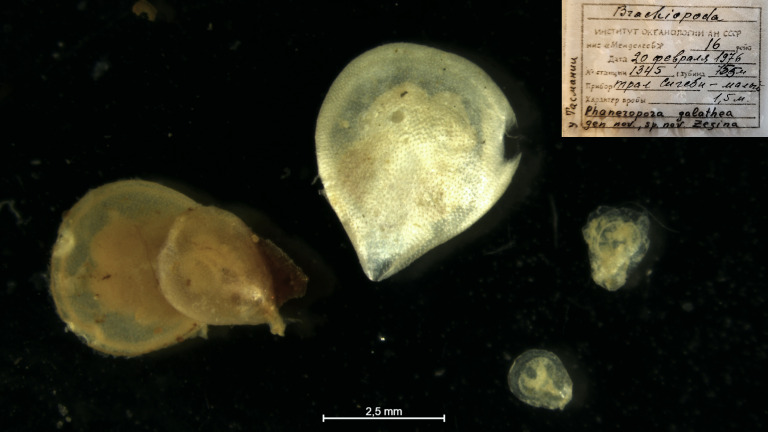
*Phaneropora galatheae* syntypes, cat. INV0008238

**Figure 6f.**
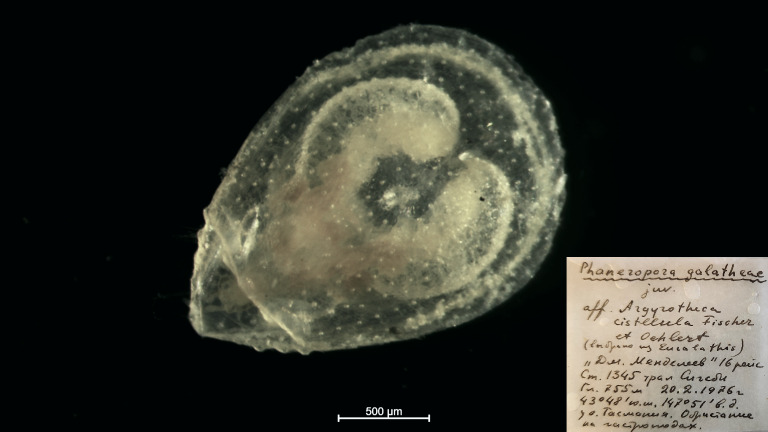
*Phaneropora galatheae* syntype, cat. INV0008239

**Figure 7a.**
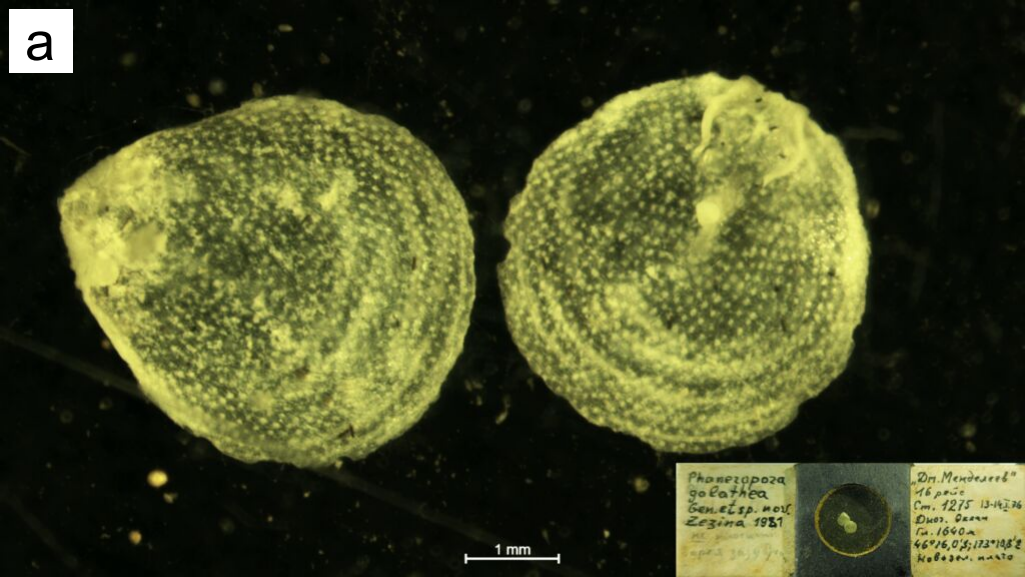
*Phaneropora galatheae* syntype, cat. INV0008252

**Figure 7b.**
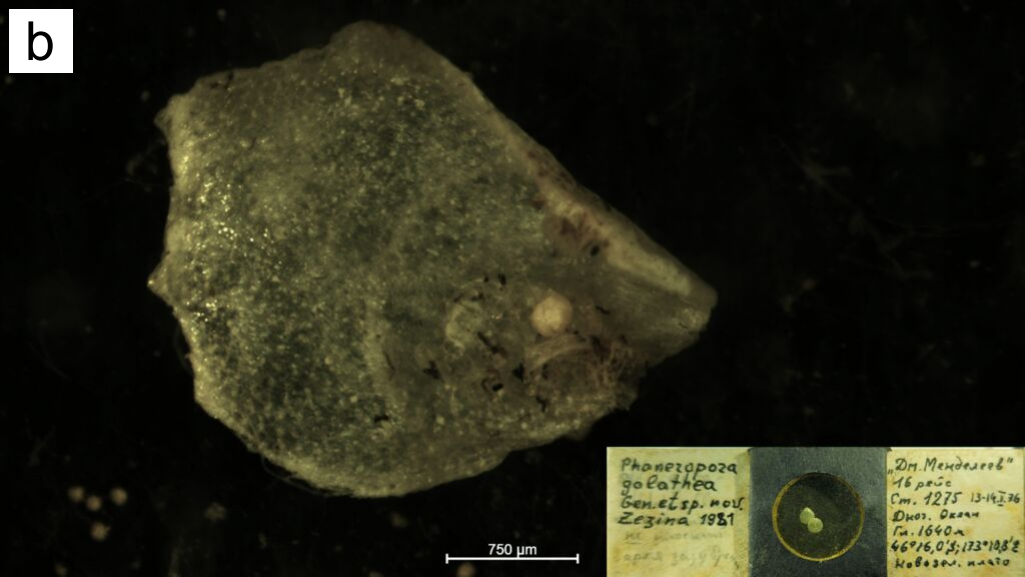
*Phaneropora galatheae* syntype, cat. INV0008253

**Figure 7c.**
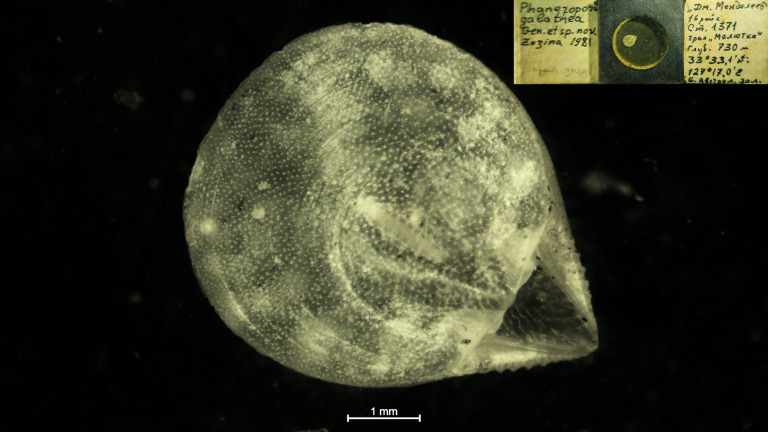
*Phaneropora galatheae* syntype, cat. INV0008254

**Figure 7d.**
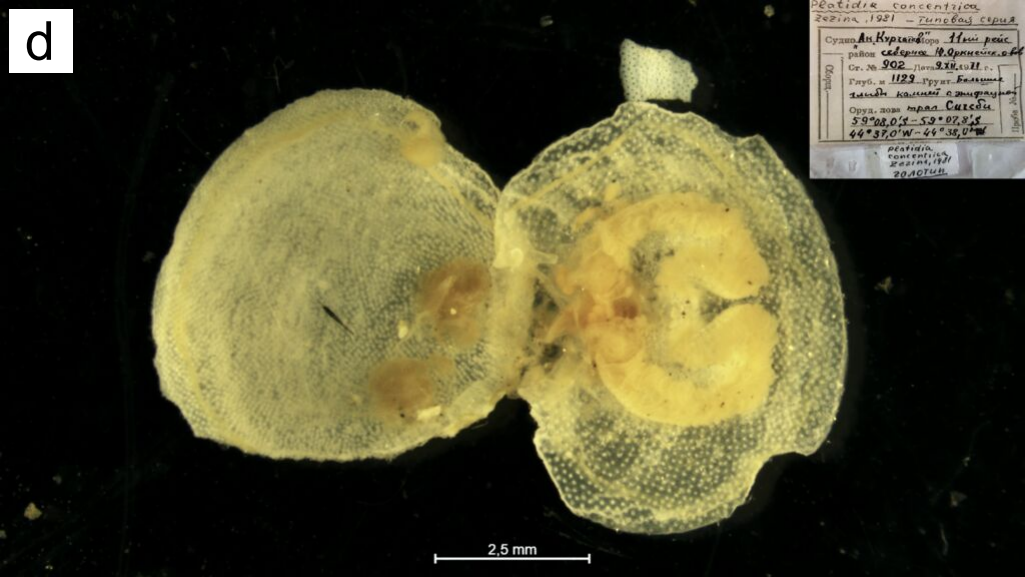
*Platidia concentrica* holotype, cat. INV0008257

**Figure 7e.**
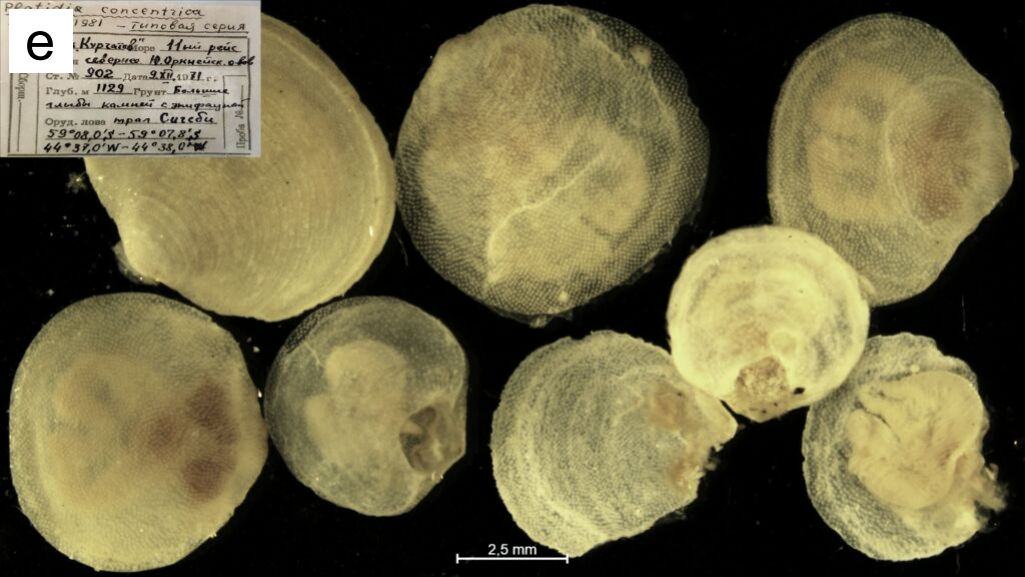
*Platidia concentrica* paratypes, cat. INV0008258

**Figure 7f.**
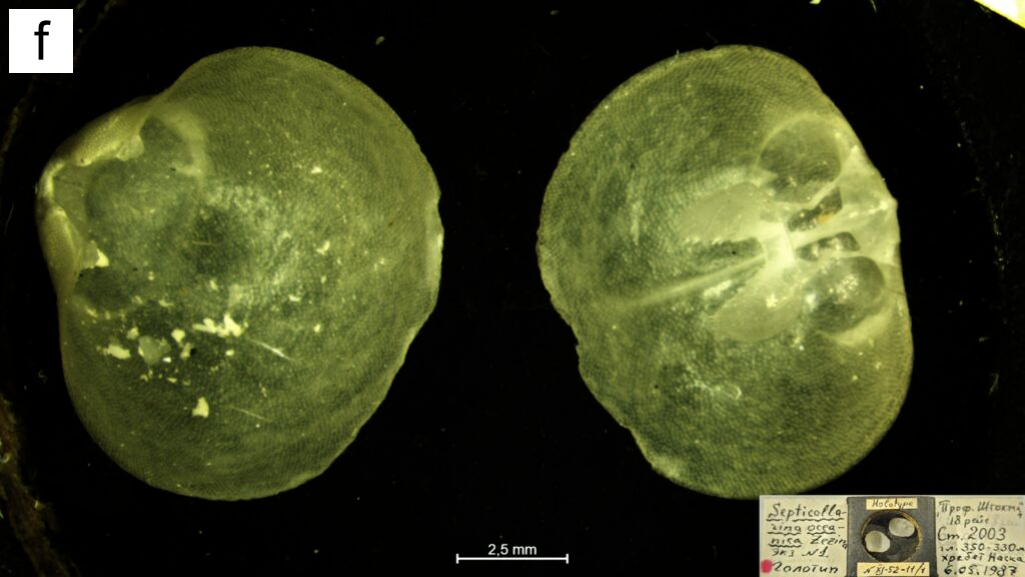
*Septicollarina oceanica* holotype, cat. INV0006027

**Figure 8a.**
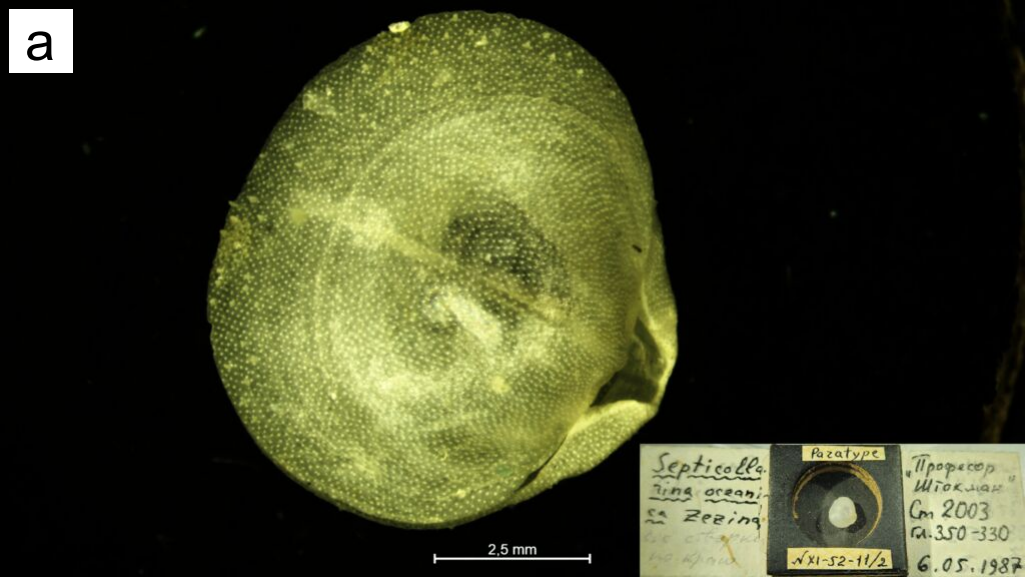
*Septicollarina oceanica* paratype, cat. INV0006028

**Figure 8b.**
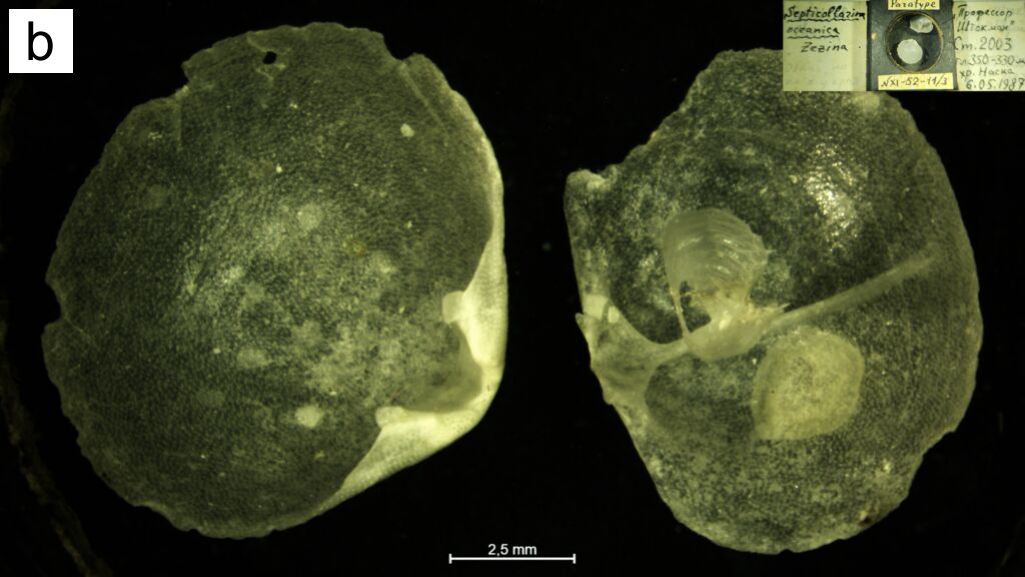
*Septicollarina oceanica* paratype, cat. INV0006029

**Figure 8c.**
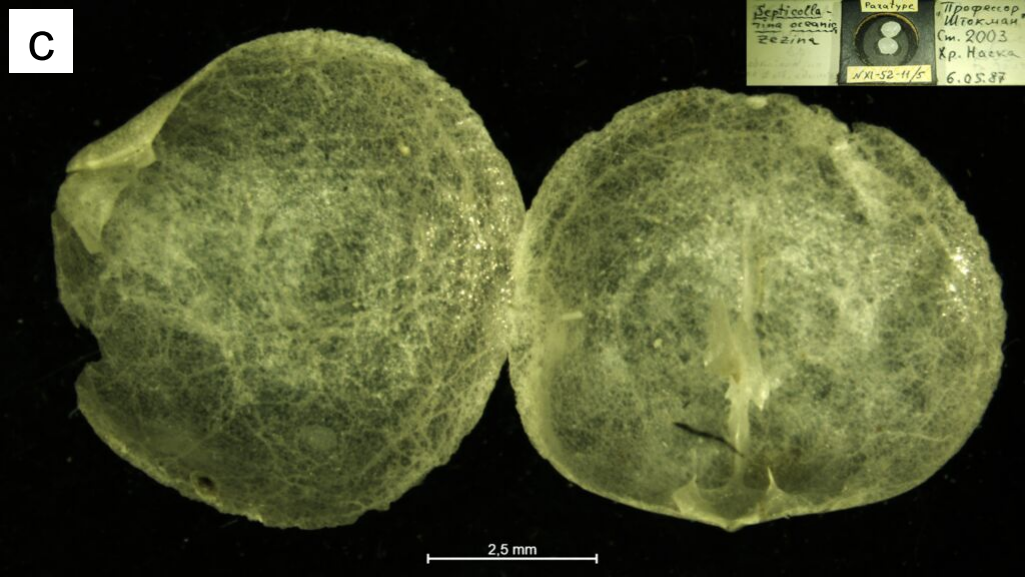
*Septicollarina oceanica* paratype, cat. INV0006030

**Figure 8d.**
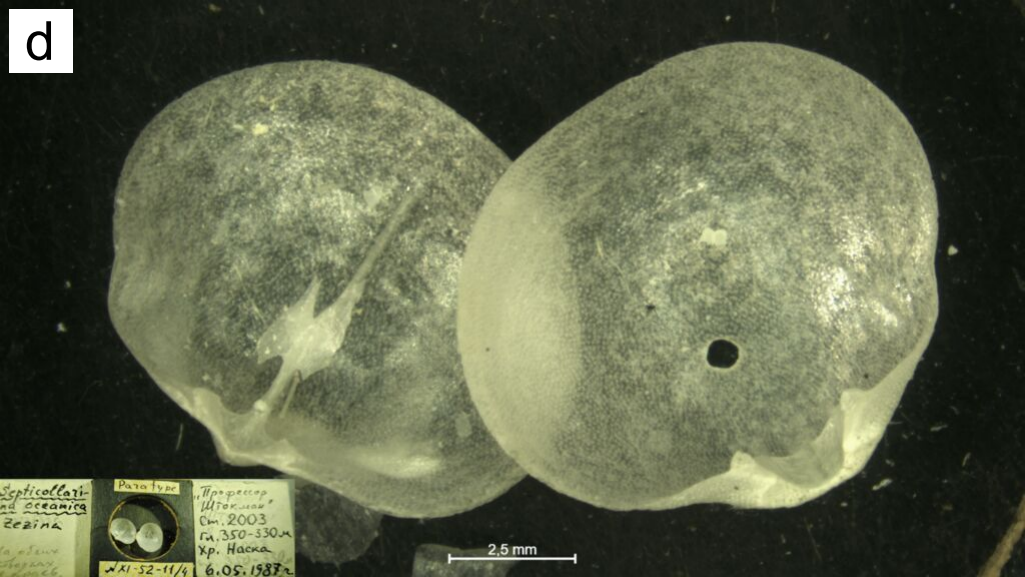
*Septicollarina oceanica* paratype, cat. INV0006031

**Figure 8e.**
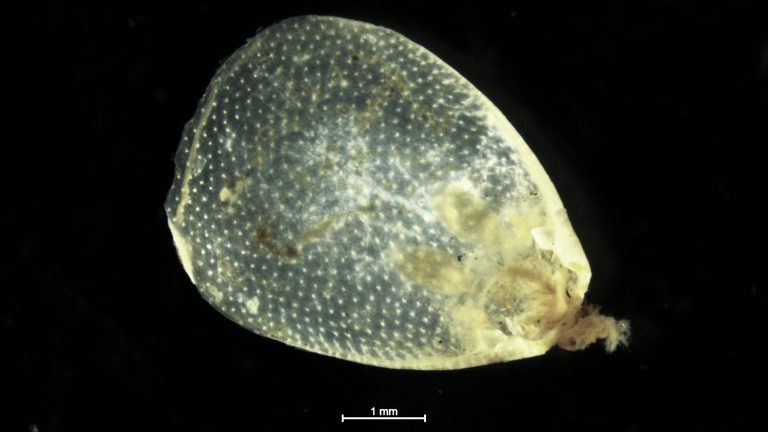
*Simpliciforma profunda* holotype, cat. INV0009010

**Figure 8f.**
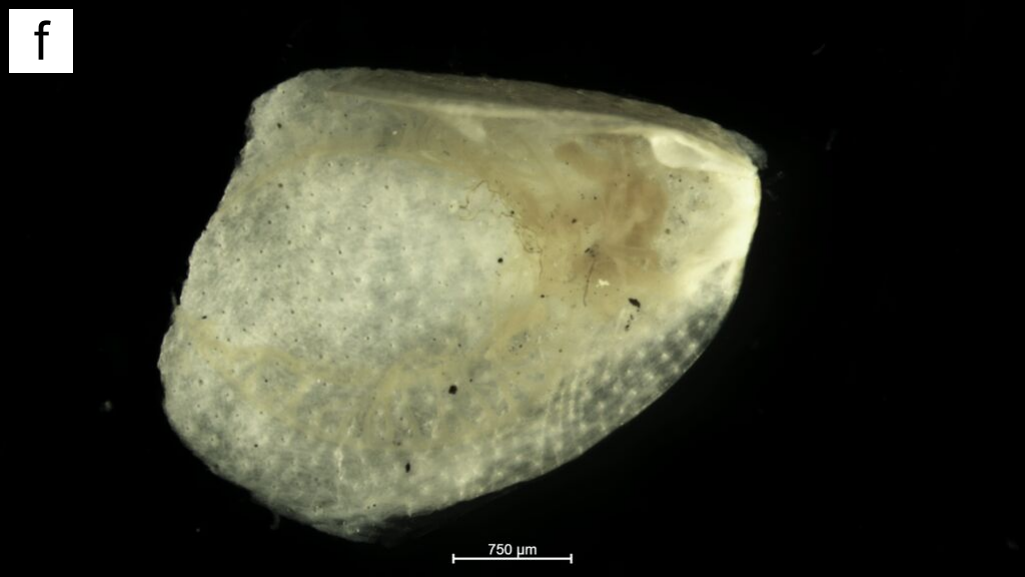
*Simpliciforma profunda* paratype, cat. INV0009011

**Figure 9a.**
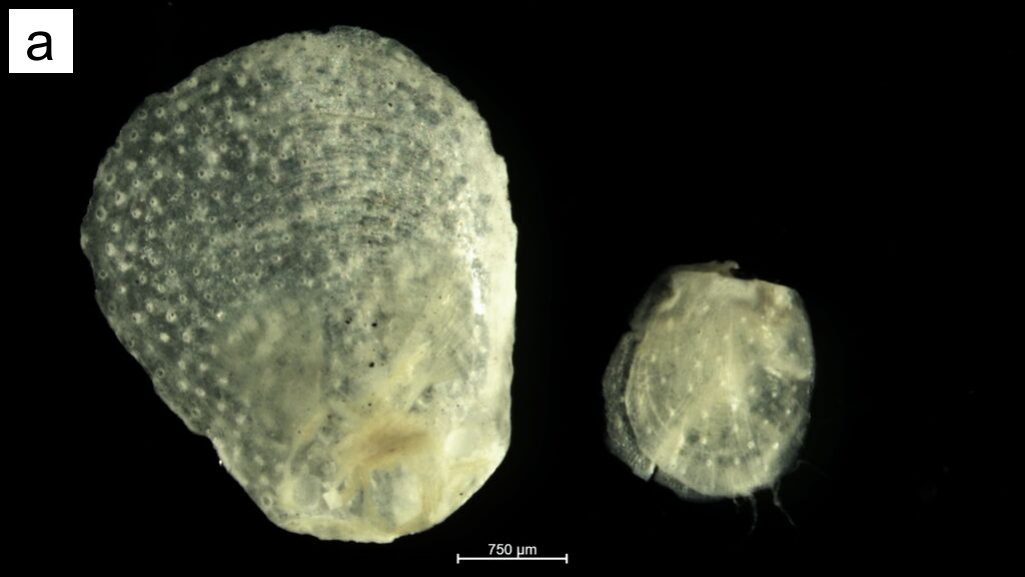
*Simpliciforma profunda* paratype, cat. INV0009012

**Figure 9b.**
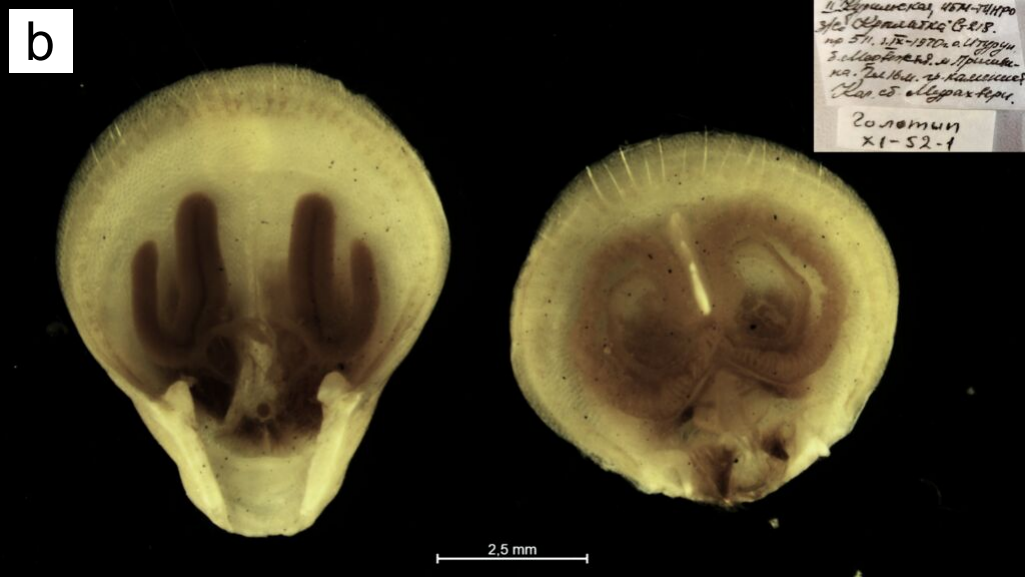
*Simplicithyris kurilensis* holotype, cat. INV0008241

**Figure 9c.**
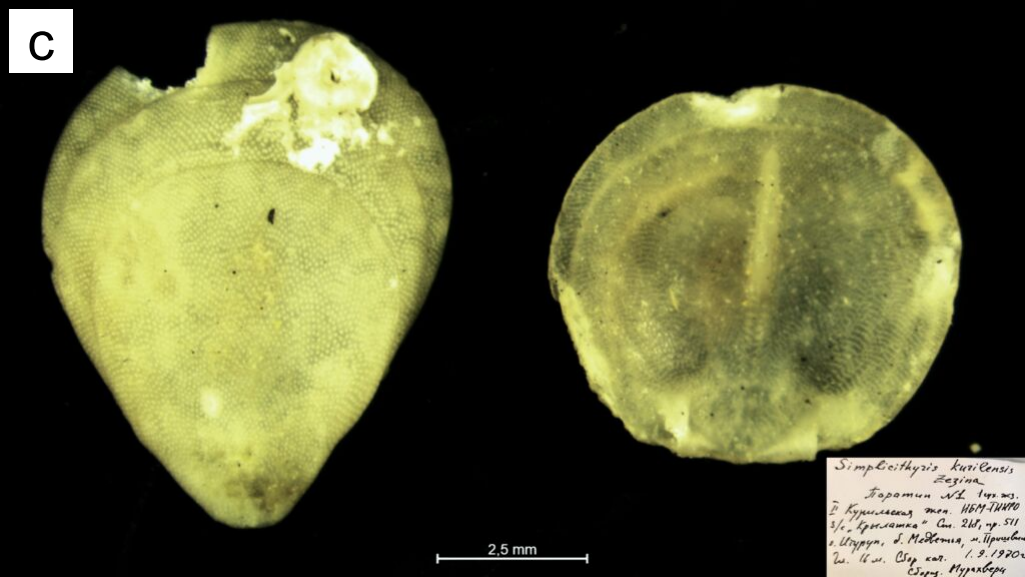
*Simplicithyris kurilensis* paratype, cat. INV0008242

**Figure 9d.**
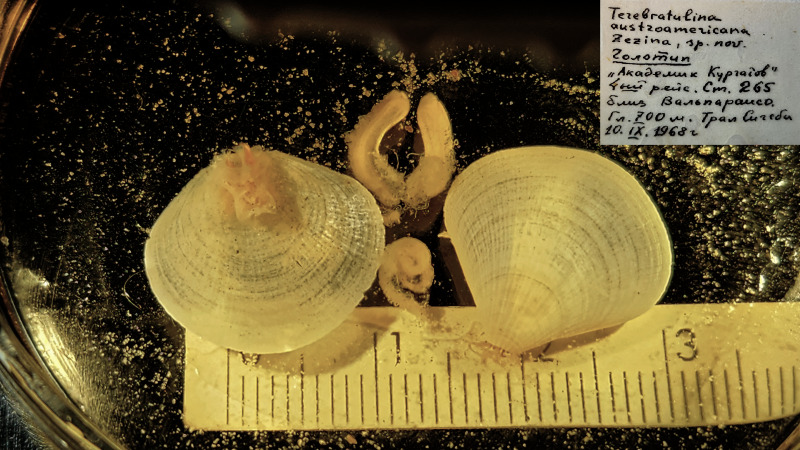
*Terebratulina austroamericana* holotype, cat. INV0006023

**Figure 9e.**
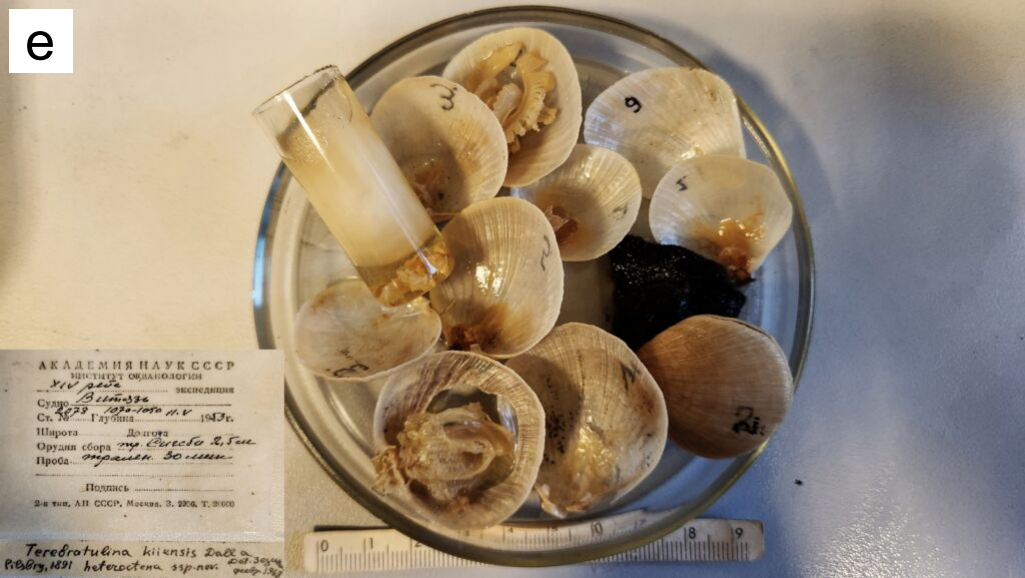
*Terebratulina kiiensis heteroctena* syntypes, cat. INV0006024

**Figure 9f.**
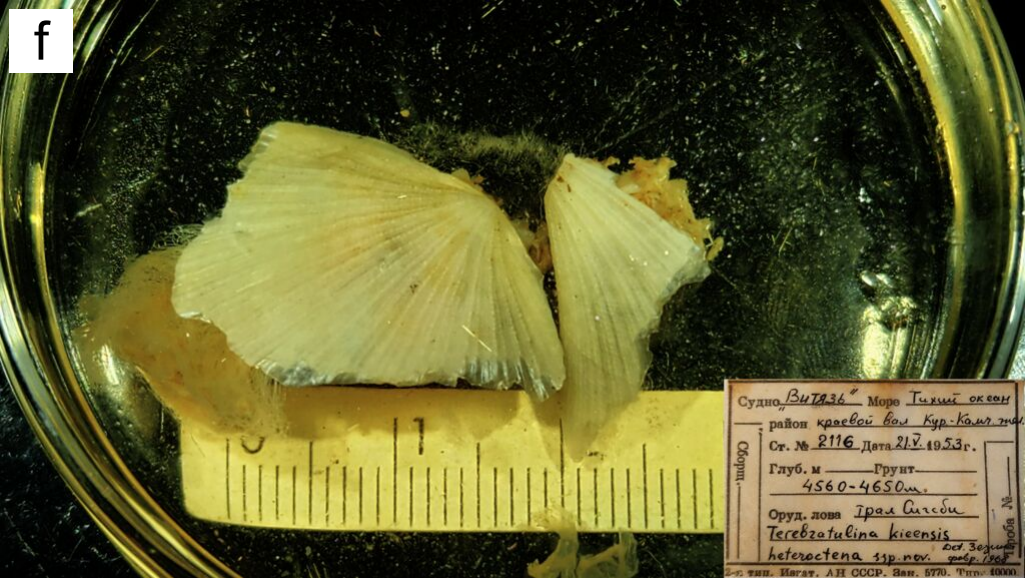
*Terebratulina kiiensis heteroctena* syntype, cat. INV0006025

**Figure 10a.**
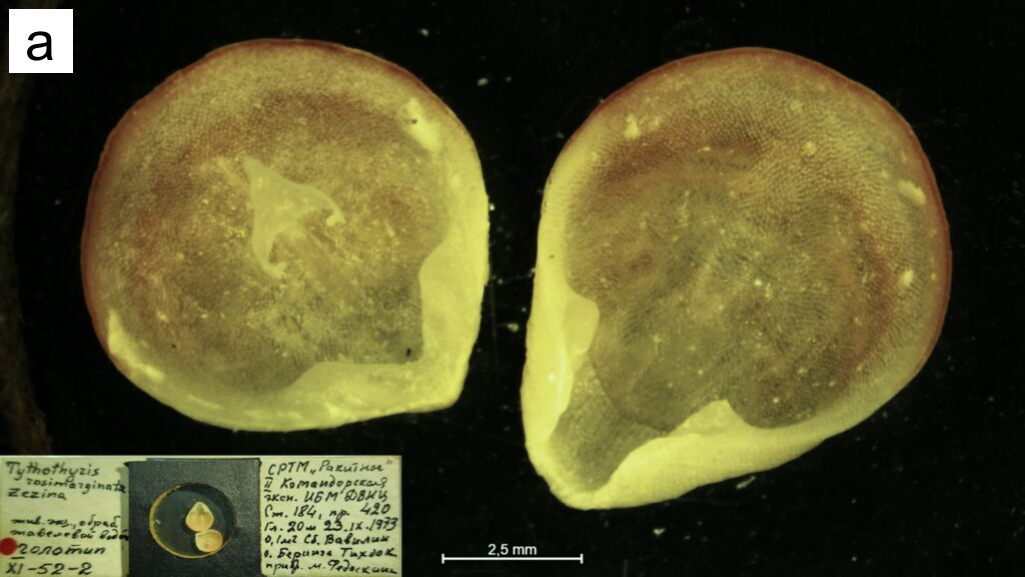
*Tythothyris rosimarginata* holotype, cat. INV0008244

**Figure 10b.**
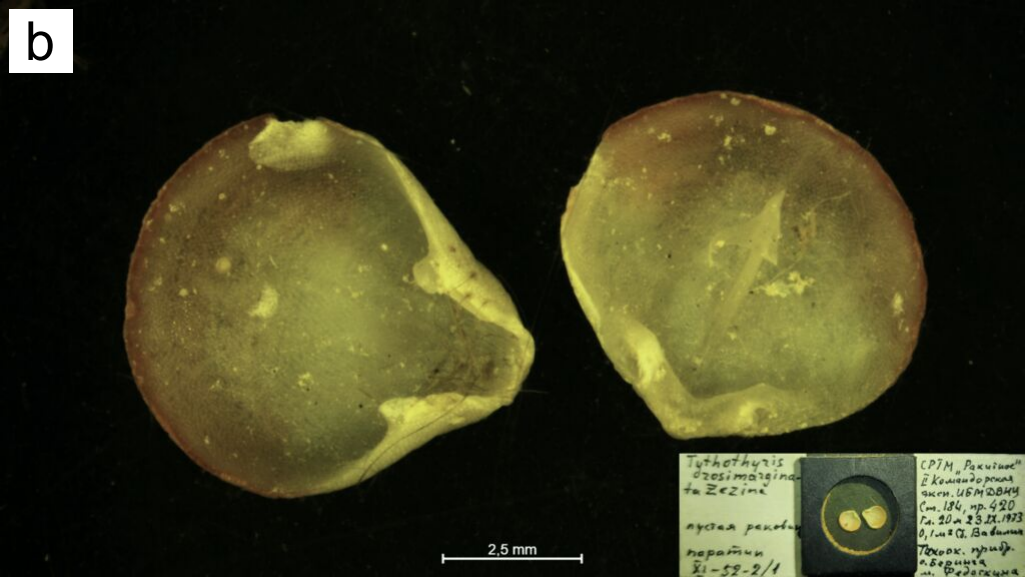
*Tythothyris rosimarginata* paratype, cat. INV0008246

**Figure 10c.**
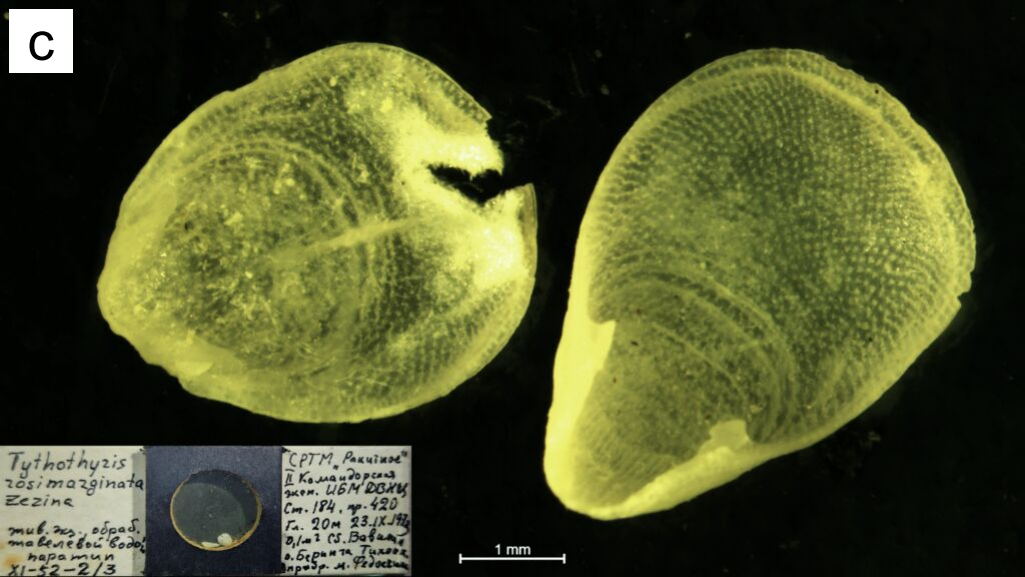
*Tythothyris rosimarginata* paratype, cat. INV0008247

**Figure 10d.**
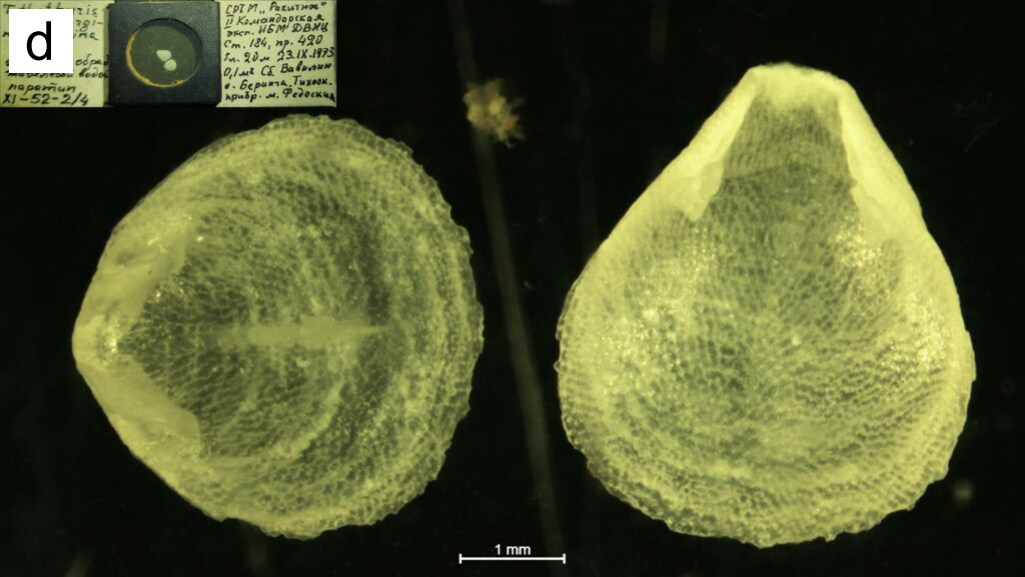
*Tythothyris rosimarginata* paratype, cat. INV0008248

**Figure 10e.**
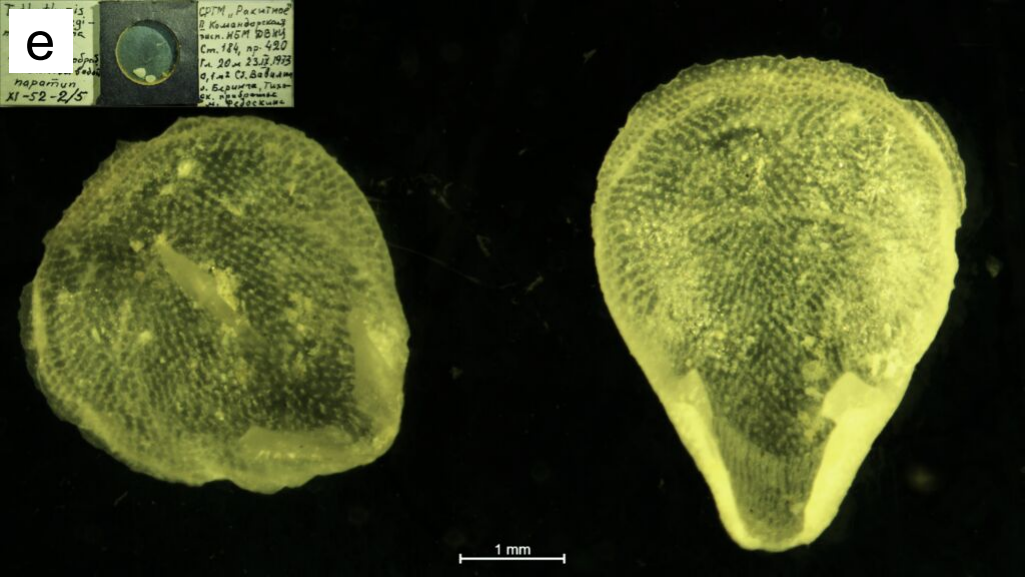
*Tythothyris rosimarginata* paratype, cat. INV0008249

**Figure 10f.**
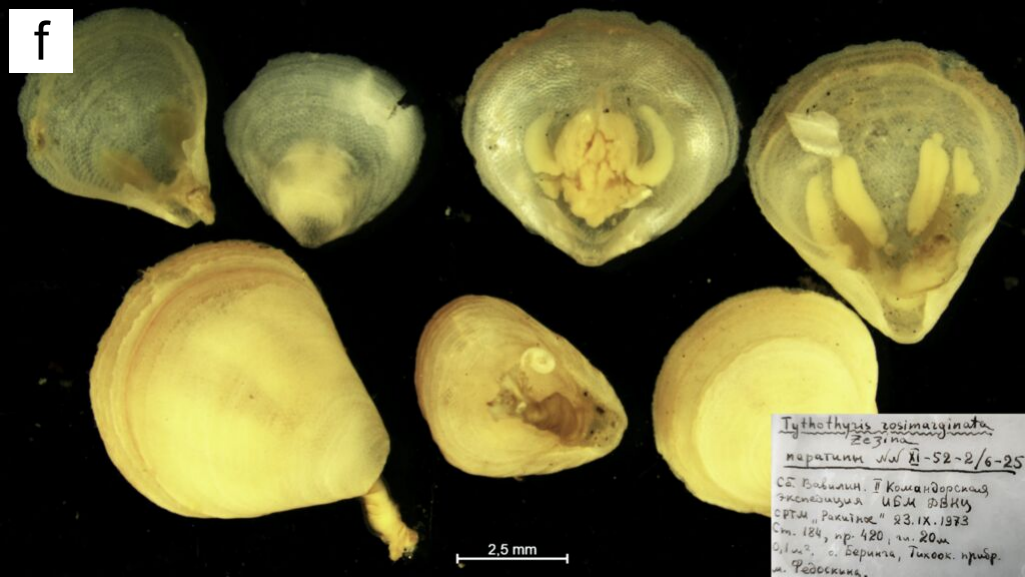
*Tythothyris rosimarginata* paratypes, cat. INV0008250

**Table 1.** Type specimens in the IORAS Brachiopoda collection. Number of paratypes/syntypes/topotypes is given in brackets after the catalogue number.

**No**.	**Scientific Name**	**Reference**	**Figure**	**Family**	**Type Status**	**Catalogue Number**
1	*Annuloplatidia indopacifica* Zezina, 1981	Zezina 1981b	Fig. 1a	Platidiidae	Holotype	INV0008255
2	*Annuloplatidia indopacifica*	Zezina 1981b	Fig. 1b	Platidiidae	Paratypes	INV0008256 (21)
3	*Bathynanus inversus* Zezina, 1981	Zezina 1981c	Fig. 1c	Chlidonophoridae	Holotype	INV0003724
4	*Bathynanus inversus*	Zezina 1981c	Fig. 1d	Chlidonophoridae	Paratypes	INV0006022 (6)
5	*Bathynanus rhizopodus* Zezina, 1981	Zezina 1981c	Fig. 1e	Chlidonophoridae	Holotype	INV0003719
6	*Bathynanus rhizopodus*	Zezina 1981c	Figs 1f, 2a	Chlidonophoridae	Paratypes	INV0003720 (1), INV0003721 (1)
7	*Cnismatocentrum sakhalinensis parvum* Zezina, 1970 (accepted as *Cnismatocentrum parvum* Zezina, 1970)	Zezina 1970b	Figs 2b, c, d, e, f, 3a, b, c	Cnismatocentridae	Syntypes	INV0006016 (1), INV0006018 (1), INV0006019 (1), INV0006020 (1), INV0006021 (1), INV0006026 (1), INV0009001 (30)
8	*Eucalathis macroctena* Zezina, 1981 (accepted as *Melvicalathis macroctena* (Zezina, 1981))	Zezina 1981c	Fig. 3d	Chlidonophoridae	Holotype	INV0003713
9	*Eucalathis macroctena*	Zezina 1981c	Fig. 3e, f	Chlidonophoridae	Paratypes	INV0003714 (1), INV0003715 (1)
10	*Grammetaria minima* Zezina, 1994	Zezina 1994	Fig. 4a	Frieleiidae	Holotype	INV0008251
11	*Grammetaria minima*	Zezina 1994	Fig. 4a	Frieleiidae	Paratypes	INV0008251 (2)
12	*Holobrachia vietnamica* Zezina, 2001	Zezina 2001	Fig. 4b		Holotype	INV0008264
13	*Holobrachia vietnamica*	Zezina 2001	Fig. 4c		Paratypes	INV0008267 (9)
14	*Holobrachia vietnamica*	Zezina 2001	Fig. 4d		Topotypes	INV0008265 (6), INV0008266 (1)
15	*Nanacalathis atlantica* Zezina,1991	Zezina 1991	Fig. 4e	Chlidonophoridae	Holotype	INV0003718
16	*Nanacalathis minuta* Zezina, 1981	Zezina 1981c	Fig. 4f	Chlidonophoridae	Holotype	INV0003716
17	*Neothyris westpacifica* Zezina, 2001	Zezina 1981c	Fig. 5a	Terebratellidae	Holotype	INV0008259
18	*Neothyris westpacifica*	Zezina 1981c	Fig. 5b, c, d	Terebratellidae	Paratypes	INV0008260 (3), INV0008261 (8), INV0008263 (24)
19	*Neothyris westpacifica*	Zezina 1981c		Terebratellidae	Topotype	INV0008262 (1)
20	*Novocrania altivertex* (Zezina, 1990)	Zezina 1990a	Fig. 5e	Craniidae	Holotype	INV0008240
21	*Novocrania altivertex*	Zezina 1990a	Fig. 5f	Craniidae	Paratypes	INV0009026 (3)
22	*Oceanithyris juveniformis* Bitner & Zezina, 2013	Bitner et al. 2013	Fig. 6a	Dyscoliidae	Holotype	INV0009005
23	*Oceanithyris juveniformis*	Bitner et al. 2013	Fig. 6b, c, d	Dyscoliidae	Paratypes	INV0009006 (1), INV0009007 (1), INV0009008 (1)
24	*Phaneropora galatheae* Zezina, 1981	Zezina 1981a	Figs 6e, f, 7a, b, c	Platidiidae	Syntypes	INV0008238 (15), INV0008239 (1), INV0008252 (1), INV0008253 (1), INV0008254 (1)
25	*Platidia concentrica* Zezina, 1980 (accepted as *Platidia anomioides* (Scacchi & Philippi in Philippi, 1844))	Zezina 1980	Fig. 7d	Platidiidae	Holotype	INV0008257
26	*Platidia concentrica*	Zezina 1980	Fig. 7e	Platidiidae	Paratypes	INV0008258 (6)
27	*Septicollarina oceanica* Zezina, 1990	Zezina 1990b	Fig. 7f	Aulacothyropsidae	Holotype	INV0006027
28	*Septicollarina oceanica*	Zezina 1990b	Fig. 8a, b, c, d	Aulacothyropsidae	Paratypes	INV0006028 (1), INV0006029 (1), INV0006030 (1), INV0006031 (1)
29	*Septicollarina oceanica*	Zezina 1990b		Aulacothyropsidae	Topotype	INV0006032
30	*Simpliciforma profunda* Bitner & Zezina, 2013	Bitner et al. 2013	Fig. 8e		Holotype	INV0009010
31	*Simpliciforma profunda*	Bitner et al. 2013	Figs 8f, 9a		Paratypes	INV0009011 (1), INV0009012 (1)
32	*Simplicithyris kurilensis* Zezina, 1976	Zezina 1976	Fig. 9b	Jagtithyrididae	Holotype	INV0008241
33	*Simplicithyris kurilensis*	Zezina 1976	Fig. 9c	Jagtithyrididae	Paratype	INV0008242 (1)
34	*Simplicithyris kurilensis*	Zezina 1976		Jagtithyrididae	Topotypes	INV0008243 (3)
35	*Terebratulina austroamericana* Zezina, 1981	Zezina 1981c	Fig. 9d	Cancellothyrididae	Holotype	INV0006023
36	*Terebratulina kiiensis heteroctena* Zezina, 1970	Zezina 1970b	Fig. 9e, f	Cancellothyrididae	Syntypes	INV0006024 (8), INV0006025 (1)
37	*Tythothyris rosimarginata* Zezina, 1979	Zezina 1979	Fig. 10a	Terebratallidae	Holotype	INV0008244
38	*Tythothyris rosimarginata*	Zezina 1979	Fig. 10b, c, d, e, f	Terebratallidae	Paratypes	INV0008246 (1), INV0008247 (1), INV0008248 (1), INV0008249 (1), INV0008250 (19)
39	*Tythothyris rosimarginata*	Zezina 1979		Terebratallidae	Topotypes	INV0007981 (8), INV0007984 (3), INV0008245 (1)

**Table 2.** Ultra-abyssal Brachiopoda specimens in the IORAS collection.

**Catalogue Number**	**Scientific Name**	**Locality**	**Latitude**	**Longitude**	**Depth**	**Collecting Event**	**Date**
INV0005341	*Pelagodiscus atlanticus* (King, 1868)	Romanche Trench	-0.23333	-18.48333	7460–7600 m	RV Akademik Kurchatov cruise 11 st.1012	16-01-1972
INV0005342	*Pelagodiscus atlanticus*	Romanche Trench	0.21667	-18.61667	7430–7500 m	RV Akademik Kurchatov cruise 11 st.1014	17-01-1972
INV0005384	*Pelagodiscus atlanticus*	Northwest Pacific	25.61833	167.88556	6160 m	RV Vityaz cruise 43 st.6012	23-04-1968

**Table 3.** Number of collection lots by genera.

**Genus**	**Nr**	**Genus**	**Nr**
*Abyssothyris* Thomson, 1927	44	*Liothyrella* Thomson, 1916	71
*Acrobrochus* Cooper, 1983	2	*Liothyrina* Oehlert, 1887	1
*Aerothyris* Allan, 1939	54	*Liothyris* DouvilIé, 1880	17
*Amphithyris* Thomson, 1918	1	*Macandrevia* King, 1859	181
*Aneboconcha* Cooper, 1973	38	*Magadina* Thomson, 1915	12
*Annuloplatidia* Zezina, 1981	7	*Magasella* Dall, 1870	104
*Argyrotheca* Dall, 1900	16	*Magellania* Bayle, 1880	31
*Basiliola* Dall, 1908	17	*Megathiris* d'Orbigny, 1847	16
*Bathynanus* Foster, 1974	17	*Megerlia* King, 1850	45
*Campages* Hedley, 1905	12	*Megerlina* Deslongchamps, 1884	3
*Cancellothyris* Thomson, 1926	10	*Melvicalathis* Lee, Lüter & Zezina in Lee, Gregory, Lüter, Zezina, Robinson & Christie, 2008	1
*Chlidonophora* Dall, 1903	51	*Minutella* Hoffmann & Lüter, 2010	1
*Cnismatocentrum* Dall, 1920	83	*Nanacalathis* Zezina, 1981	6
*Compsothyris* Jackson, 1918	8	*Neoancistrocrania* Laurin, 1992	1
*Coptothyris* Jackson, 1918	2	*Neorhynchia* Thomson, 1915	16
*Crania* Retzius, 1781	46	*Neothyris* Douvillé, 1879	24
*Cryptopora* Jeffreys, 1869	20	*Nipponithyris* Yabe & Hatai, 1934	1
*Dallina* Beecher, 1893	26	*Notosaria* Cooper, 1959	3
*Dallinella* Thomson, 1915	2	*Notozyga* Cooper, 1977	4
*Dallithyris* Muir-Wood, 1959	36	*Novocrania* Lee & Brunton, 2001	13
*Diestothyris* Thomson, 1916	275	*Obolus* Eichwald, 1829	1
*Discina* Lamarck, 1819	2	*Oceanithyris* Bitner & Zezina, 2013	5
*Discinisca* Dall, 1871	4	*Orbicula* Cuvier, 1798	1
*Discradisca* Stenzel, 1964	2	*Ospreyella* Lüter & Wörheide, 2003	1
*Dolichozygus* Cooper, 1983	1	*Parasphenarina* Motchurova-Dekova, Saito & Endo, 2002	1
*Dyscolia* Fischer & Oehlert, 1890	2	*Pelagodiscus* Dall, 1908	276
*Eohemithiris* Hertlein & Grant, 1944	1	*Phaneropora* Zezina, 1981	24
*Eucalathis* Fischer & Oehlert, 1890	32	*Platidia* Costa, 1852	52
*Fallax* Atkins, 1960	5	*Pseudocrania* McCoy, 1851	1
*Fosteria* Zezina, 1980	5	*Pumilus* Atkins, 1958	1
*Frenulina* Dall, 1895	3	*Rhynchonella* Fischer de Waldheim, 1809	16
*Frieleia* Dall, 1895	40	*Septicollarina* Zezina, 1981	6
*Glaciarcula* Elliott, 1956	78	*Shimodaia* MacKinnon, Saito & Endo, 1997	1
*Glottidia* Dall, 1870	9	*Simpliciforma* Bitner & Zezina, 2013	3
*Grammetaria* Cooper, 1959	1	*Simplicithyris* Zezina, 1976	48
*Gryphus* Megerle von Mühlfeld, 1811	29	*Tegulorhynchia* Chapman & Crespin, 1923	1
*Gwynia* King, 1859	8	*Terebratalia* Beecher, 1893	50
*Gyrothyris* Thomson, 1918	9	*Terebratella* d'Orbigny, 1847	60
*Hemithiris* d'Orbigny, 1847	296	*Terebratula* Müller, 1776	4
*Hispanirhynchia* Thomson, 1927	20	*Terebratulina* d'Orbigny, 1847	271
*Holobrachia* Zezina, 2001	4	*Tethyrhynchia* Logan in Logan & Zibrowius, 1994	2
*Jaffaia* Thomson, 1927	7	*Thaumatosia* Cooper, 1973	1
*Jolonica* Dall, 1920	2	*Thecidellina* Thomson, 1915	5
*Laqueus* Dall, 1870	174	*Tythothyris* Zezina, 1979	101
*Leptothyrella* Muir-Wood, 1965	2	*Waldheimia* King, 1850	1
*Leptothyris* Muir-Wood, 1959	3	*Waltonia* Davidson, 1850	4
*Lingula* Bruguière, 1791	3	*Xenobrochus* Cooper, 1981	2
